# Influence of *MTHFR* polymorphism, alone or in combination with smoking and alcohol consumption, on cancer susceptibility

**DOI:** 10.1515/biol-2022-0680

**Published:** 2023-09-26

**Authors:** Yonghui Huang, Qiurui Hu, Zhenxia Wei, Li Chen, Ying Luo, Xiaojie Li, Cuiping Li

**Affiliations:** Department of Prosthodontics, The Affiliated Stomatology Hospital of Guangxi Medical University, Nanning 530021, P. R. China; Department of Oral and Maxillofacial Surgery, The Affiliated Stomatology Hospital of Guangxi Medical University, Nanning 530021, P. R. China; Medical Scientific Research Center, College of Stomatology, Guangxi Medical University, Nanning 530021, P. R. China; Guangxi Key Laboratory of Oral and Maxillofacial Rehabilitation and Reconstruction, Guangxi Clinical Research Center for Craniofacial Deformity, Guangxi Health Commission Key Laboratory of Prevention and Treatment for Oral Infectious Diseases, Nanning 530021, P. R. China; Department of Experiment, The Affiliated Stomatology Hospital of Guangxi Medical University, Nanning 530021, P. R. China

**Keywords:** *MTHFR*, cancer, polymorphism, meta-analysis

## Abstract

5,10-methylenetetrahydrofolate reductase (MTHFR) mutations play a significant role in various types of cancers, serving as crucial regulators of folate levels in this process. Several studies have examined the effects of smoking and drinking on MTHFR-related cancers, yielding inconsistent results. Therefore, the objective of this study was to evaluate the magnitude of the effects of gene-smoking or gene-drinking interactions on cancer development. We conducted a comprehensive literature search in PubMed, Web of Science, CNKI, and Wan Fang databases up until May 10th, 2022, to identify relevant articles that met our inclusion criteria. The extracted data from these studies were used to calculate the overall odds ratio (OR) and corresponding 95% confidence interval (95% CI) using either a fixed-effect or random-effect model in Stata version 11.2. Stratified analyses were performed based on ethnicity, control group origin, and cancer classification to assess the risk of cancers associated with gene-smoking or gene-drinking interactions. Sensitivity analyses were conducted to investigate potential sources of heterogeneity, and publication bias was assessed using the Begg’s test and Egger’s test. Additionally, regression analysis was employed to explore the influence of relevant variables on heterogeneity. To evaluate the statistical correlations, analytical methods such as the false-positive report probability and the Bayesian false discovery probability were applied to assess the reliability of the findings. In our meta-analysis, a total of 47 articles were included, comprising 13,701 cases and 21,995 controls for the C677T polymorphism and 5,149 cases and 8,450 controls for the A1298C polymorphism. The results indicated a significant association between C677T polymorphism and cancer risks when combined with smoking (CT + TT vs CC, OR [95% CI] = 1.225 [1.009–1.487], *p* = 0.041). Stratified analysis further revealed a significant increase in liver cancer risk for individuals with the C677T when combined with smoking (liver cancer: CT + TT vs CC, OR [95% CI] = 1.564 [1.014–2.413], *p* = 0.043), particularly among Asian smokers (CT + TT vs CC, OR [95% CI] = 1.292 [1.007–1.658], *p* = 0.044). Regarding the A1298C polymorphism, an elevated risk of cancer was observed in mixed populations alone (CC + AC vs AA, OR [95% CI] = 1.609 [1.087–2.381], *p* = 0.018), as well as when combined with smoking (CC + AC vs AA, OR [95% CI] = 1.531 [1.127–2.080], *p* = 0.006). In non-drinkers, C677T polymorphism was found to be associated with esophageal cancer risk (C677T: CT + TT vs CC, OR [95% CI] = 1.544 [1.011–2.359], *p* = 0.044) and colon cancer risk (CC + AC vs AA, OR [95% CI] = 1.877 [1.166–3.054], *p* = 0.010), but there was no clear link between this polymorphism and cancer risk among drinkers. The association between the C677T polymorphism and cancer risk among smokers was found to be significant, suggesting that the combination of tobacco and the C677T polymorphism may enhance the carcinogenic process, particularly in liver cancer. However, no similar relationship was observed for the A1298C polymorphism. Interestingly, significantly increased cancer risk was observed in individuals with C677T genetic variants who were nondrinkers, but not among drinkers. These findings highlight the potential role of the C677T polymorphism in modifying cancer risk in specific contexts, such as smoking and alcohol consumption.

## 1 Introduction

Cancer is a serious human disease that has led to high mortality rates worldwide. Nowadays, increasing evidence suggests that cancer development is the result of the combined effect of environmental and genetic factors. Single nucleotide polymorphism (SNP) is the most common and stable genetic variants, and germline sequence variation could be discovered in more than 1% of the general population, which might account for 80% of the whole genome heterogeneity [1]. Based on the fundamental structure changes, researchers use SNPs expediently for genotyping to determine whether they are directly or indirectly linked to specific traits or diseases [2].

Folic acid (vitamin B9) is a crucial micronutrient that cannot be synthesized by the body and is found in dark green leafy vegetables and legumes [3]. Through the action of dihydrofolate (DHF) reductase, folic acid is converted to DHF, which is then further reduced to tetrahydrofolate (THF). THF is transformed into 5,10-methylenetetrahydrofolate (5,10-MTHF). Under the influence of the MTHF reductase (MTHFR) enzyme, 5,10-MTHF is converted to 5-MTHF, which acts as a methyl donor in the synthesis of pyrimidines and purines. Additionally, in the methylation pathway, 5-MTHF plays a critical role by converting homocysteine (Hcy) into S-adenosylmethionine (SAM). SAM is instrumental in DNA methylation processes [4].

MTHFR is one of the most extensively studied genes, playing a crucial role in folate metabolism [5]. MTHFR is located at the short arm terminus of chromosome 1 (1p36.3). The DNA sequence of this MTHFR is approximately 2.2 kb (kilobases) long and consists of 11 exons [6]. The C677T (rs1801133) and A1298C (rs1801131) are the most extensively studied mutations in the MTHFR variants [7]. They are located in exons 4 and 7, respectively. Both variations are associated with a reduction in enzyme activity [8,9]. C677T variation is present in approximately 20–40% of the population. Research findings indicate that individuals with heterozygous (CT) and homozygous mutant (TT) genotypes exhibit enzyme activities reduced to 65% and 30%, respectively, in comparison to the wild-type individuals (CC), under the C → T condition [10]. This variant involves the conversion of cytosine to thymine, leading to the substitution of methionine with valine at amino acid position 222 in the MTHFR enzyme structure [11]. Additionally, this mutation introduces an HinfI restriction site. As a result, the enzyme’s temperature stability is compromised, causing its efficiency to decrease by approximately half and resulting in elevated levels of Hcy in individuals with low folate intake [12]. The A1298C polymorphism is located 2.1 kb downstream of C677T and results in an A to C conversion at codon 429, leading to the substitution of glutamine with alanine in the MTHFR protein. This ultimately results in decreased enzyme activity. However, compared to the C677T homozygous genotype (TT) that significantly increases plasma Hcy levels, the 1298CC genotype does not show a significant increase in Hcy levels [13].

Tobacco smoke contains a substantial quantity of carcinogens and toxic substances that have the potential to promote the development of cancer and other diseases [14]. These harmful components can contribute to conditions such as asthma and trigger inflammatory responses within the body. Additionally, tobacco smoke has the ability to induce genetic mutations, playing a significant role in the underlying mechanisms of tobacco-related illnesses [15]. Scientific studies have demonstrated that smoking can result in double-stranded DNA breaks, which, if left unrepaired, significantly increase the individual’s risk of cancer. Moreover, smoking also influences the levels of Hcy in the body, leading to alterations in global DNA methylation [16].

Alcohol (ethanol) is also considered a carcinogen, and acetaldehyde, a metabolite of ethanol, can induce inflammation in the body, leading to the generation of reactive oxygen species and subsequent downstream effects. Moreover, acetaldehyde exhibits high reactivity with DNA and possesses various carcinogenic and genotoxic properties [17]. Due to its high reactivity with DNA, acetaldehyde can potentially form DNA adducts, altering its physical properties and interfering with DNA synthesis and repair, which is one of the major contributing factors in its carcinogenic mechanism.

With a further understanding of *MTHFR* polymorphisms, numerous studies have been carried out to explore the relationship between *MTHFR* polymorphisms and cancers. However, the specific interactions between these polymorphisms and smoking or drinking have not been thoroughly evaluated. Therefore, we conducted a comprehensive meta-analysis using the largest available sample size to investigate the impact of MTHFR SNPs on cancers in conjunction with smoking or drinking. To the best of our knowledge, no previous study has systematically examined the association between MTHFR variants, smoking or drinking, and various types of cancers. We anticipate that this meta-analysis will provide valuable insights into the field of cancer prevention.

## 2 Material and methods

### 2.1 Literature search strategy and selection criteria

The relevant medical literature was retrieved from PubMed, Web of Science, CNKI, and Wan Fang electronic databases using various combinations of search terms, including “smoking,” “tobacco,” “cigarette,” “drinking,” “alcohol,” “C677T,” “A1298C,” “rs1801133,” “1801131,” “MTHFR,” “Methylenetetrahydrofolate reductase,” and “cancer.” The search was conducted until May 10th, 2022. To ensure the inclusion of as many relevant studies as possible, we manually screened the references of other reviews, including meta-analyses. The search process was performed multiple times to ensure that no additional articles were missed. Both Chinese and English articles were considered in our analysis.

### 2.2 Inclusive and exclusive criteria

The selection criteria for eligible studies were as follows: (a) original and case-control studies; (b) examining the relationship between the C677T or A1298C polymorphism and cancer risk; (c) Articles were included in our study if they reported smoking and drinking status categorized as either “never” and “current/ever” or as “nonsmoker/nondrinker” and “smoker/drinker”; (d) providing original data for calculating the crude odds ratio (OR) and 95% confidence interval (CI) among smokers and drinkers; and (e) in cases where multiple articles reported the same primary data by the same author, only the most recent one was considered.

The articles excluded were based on the following criteria: (a) duplicate and overlapped studies; (b) case-only studies; (c) reviews (including meta-analysis), as well as meeting abstracts; (d) studies uncorrelated with one of *MTHFR* polymorphisms (C677T and A1298C); (e) The classification of smoking and drinking data did not meet our requirements: For example, if smoking was only classified by pack-years and alcohol consumption was only classified by the amount consumed, and if there was a lack of genetic typing data available for those with smoking or drinking status, then they were not included in the analysis.

### 2.3 Data extraction

The raw data were independently extracted from the included studies by two investigators: including the last name of the first author, year of publication, country, ethnicity, cancer types, control group origin, the sample size of genotypes among smokers and drinkers, and genotyping method. The extracted data were then reviewed by other researchers to ensure accuracy. In case of any discrepancies during the data extraction process, all authors discussed and reached a consensus. For subgroup analysis, the ethnicity was generally categorized into three groups: Asian, Caucasian, and Mixed. Control group origin was divided into two groups: hospital-based and population-based. Extractive studies that provided information on smoking or drinking classification (never, current/ever) were used to further evaluate the impact of these variables on cancer in relation to the polymorphisms.

### 2.4 Statistical analysis

The association between C677T or A1298C polymorphism and cancer risks among smokers and drinkers was evaluated using crude ORs and 95% CIs. Various models were employed to calculate the overall OR in MTHFR polymorphisms, specifically homozygote comparison (TT vs CC; CC vs AA), heterozygote comparison (CT vs CC; AC vs AA), dominant model (TT + CT vs CC; CC + AC vs AA), and recessive model (CC vs CT + TT; AA vs AC + CC). Subgroup analyses were conducted to examine the effects of these polymorphisms based on ethnicity, source of control, and cancer types.

To assess heterogeneity across studies, two statistical indices, namely *I* ^2^ and chi-square *p*-value, were utilized, taking into account the variation in sample sizes. If *I* ^2^ > 50% and chi-square *p*-value ≤0.05, indicating significant heterogeneity among studies, a random-effects model was used to estimate the combined OR and 95% CI. Otherwise, a fixed-effect model was employed.

Meta-regression analyses were conducted to investigate potential sources of heterogeneity that could influence the results, taking into account the year of publication, ethnicity, and control group origin. Sensitivity analyses were carried out to assess the robustness of the findings by systematically removing individual studies and examining their impact on the overall results. Publication bias was assessed using the Begg funnel plot, which plots the standard error against the log (OR), and the Egger regression asymmetry test. All statistical analyses were performed using Stata software (version 11.2). A *p*-value below 0.05 was considered statistically significant, and all *p*-values were two-sided.

To assess the credibility of significant associations, we employed the false-positive report probability (FPRP) and Bayesian false discovery probability (BFDP) as calculation methods. We set a pre-established threshold of 0.2 for FPRP and considered values below this threshold as noteworthy. Additionally, we used prior probabilities of 0.25, 0.1, 0.01, 0.001, and 0.0001 to determine the association between an odds ratio (OR) of 1.5 and cancer risk. Furthermore, we set a threshold of 0.8 for BFDP, and considered values below this threshold as noteworthy. Prior probabilities of 0.01, 0.001, and 0.00001 were used in the BFDP calculations. Similarly, we set 0.8 as the thresholds of BFDP and took 0.01, 0.001, 0.00001 as the prior probability of BFDP. It is important to note that FPRP values below 0.2 and BFDP values below 0.8 were considered significant and deserving of attention [18,19].

## 3 Result

### 3.1 Identification and characteristic of studies

**Figure 1:**
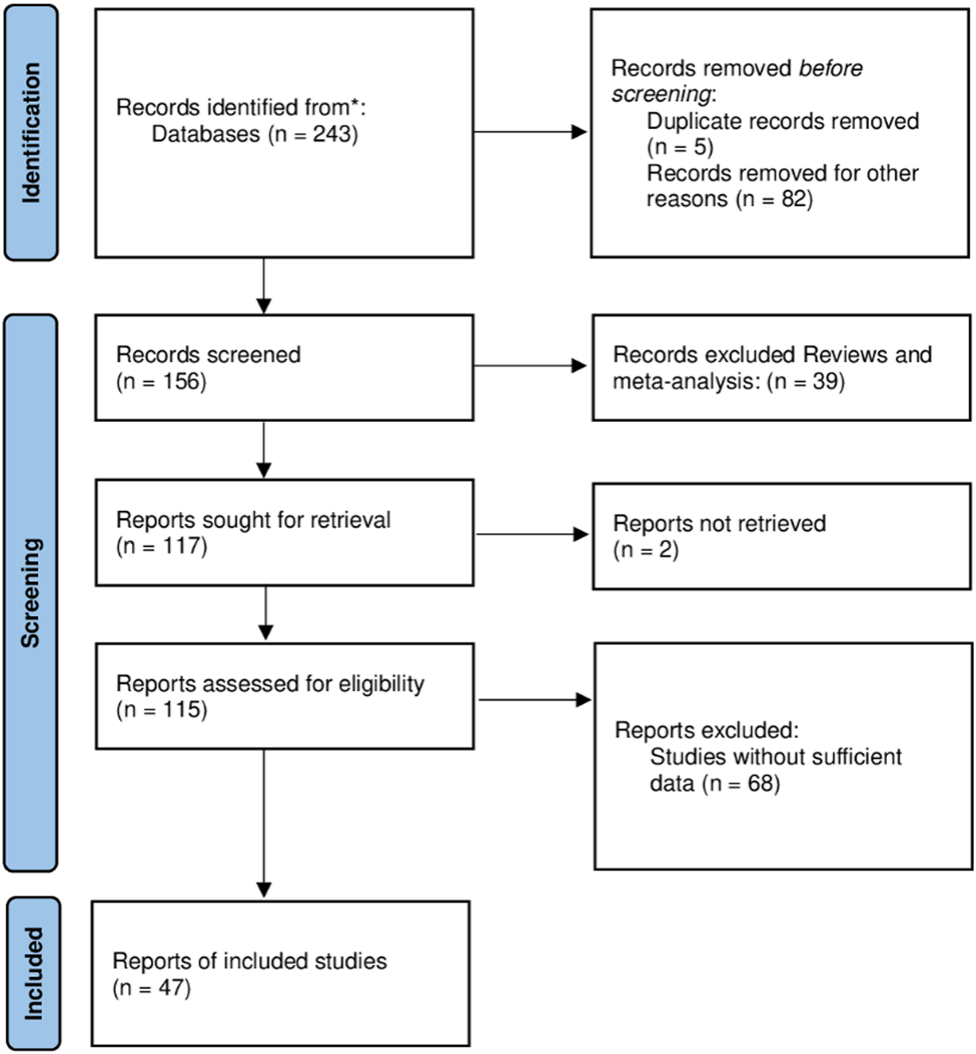
The entire flow diagram of filtering the available articles in this Study.

As depicted in Figure 1, our search strategy yielded a total of 243 studies. Through an initial screening of titles and abstracts, we excluded 82 studies that were deemed irrelevant, as well as 39 reviews, including meta-analyses, based on the exclusion criteria. Subsequently, upon careful examination of the full text of each article, we excluded 5 studies due to data overlap or duplication, 2 studies without complete full text access, and 68 studies that lacked sufficient data for calculation. Ultimately, our analysis included a total of 47 studies, comprising 44 studies with 13,701 cases and 21,995 controls for C677T polymorphism [20,21,22,23,24,25,26,27,28,29,30,31,32,33,34,35,36,37,38,39,40,41,42,43,44,45,46,47,48,49,50,51,52,53,54,55,56,57], 19 studies with 5,149 cases and 8,450 controls for A1298C polymorphism [20,22,23,25,27,31,33,36,39,41,43,44,45,46,48,58,59,60] (Tables 1 and 2).

**Table 1:** Characteristics of the eligible studies for C677T polymorphism among smokers, non-smokers, drinkers, and non-drinkers

No.	Author	Year	Ethnicity	Country	Cancer type	Source of control	Sample size of case	Sample size of control	Smoking		Non-smoking		Drinking		Non-drinking		
									Case: CC/CT/TT	Control: CC/CT/TT	Case: CC/CT/TT	Control: CC/CT/TT	Case: CC/CT/TT	Control: CC/CT/TT	Case: CC/CT/TT	Control: CC/CT/TT	Genotyping Method
1	Han	2020	Asian	China	Gastric cancer	HB	307	560	55/54/13	43/94/21	72/86/27	121/201/80	42/35/8	38/78/18	85/105/32	126/217/83	SNP
2	Ding	2018	Asian	China	Lung cancer	PB	521	1,030	88/98/18	89/94/19	153/137/26	352/372/103	31/38/8	30/40/11	210/197/36	411/197/36	SNPscan
3	Álvarez-Avellón	2017	Caucasian	Spain	Lung cancer	HB	871	825	NA	NA	NA	NA	187/190/66	201/218/63	45/57/22	61/48/11	SNP
4	Nakao	2016	Asian	Japan	Pancreatic caner	HB	360	400	79/100/36	61/105/32	48/61/36	63/89/50	80/103/43	70/128/55	47/58/29	54/66/27	SNP-type
5	Peres	2016	Mixed	Brazil	Liver cancer	HB	71	356	13/26^a^	64/93^a^	15/17^a^	85/114^a^	14/29^a^	64/102^a^	14/14^a^	85/105^a^	PCR-RFLP
6	Zara-Lopes	2016	Mixed	Brazil	Thyroid cancer	PB	100	144	34/4^b^	36/2^b^	34/4^b^	95/11^b^	16/4^b^	38/3^b^	65/15^b^	93/10^b^	PCR-RFLP
7	Zara-Lopes	2016	Mixed	Brazil	Breast cancer	PB	100	144	28/8^b^	36/2^b^	55/9^b^	95/11^b^	35/11^b^	38/3^b^	48/6^b^	93/10^b^	PCR-RFLP
8	Svensson	2016	Asian	Japan	Colorectal cancer	PB	356	709	NA	NA	NA	NA	87/61/28	90/135/33	86/84/30	151/190/81	SNP
9	Galbiatti	2011	Mixed	Brazil	Head and neck cancer	HB	322	531	108/155^a^	86/129^a^	29/31^a^	140/176^a^	90/133^a^	104/158^a^	40/60^a^	122/147^a^	PCR-RFLP
10	Tsai	2011	Asian	Taiwan	Oral cancer	PB	620	620	303/142/32	241/179/47	88/44/11	81/57/15	NA	NA	NA	NA	PCR
11	Zhao	2011	Asian	China	Esophageal cancer	HB	155	310	46/36^a^	95/38^a^	22/53^a^	85/93^a^	51/62^a^	94/74^a^	17/25^a^	85/57^a^	PCR
12	Arslan	2010	Asian	China	Lung cancer	PB	64	61	25/23/6	24/20/2	5/4/1	5/9/1	NA	NA	NA	NA	multiple PCR
13	Cao	2010	Asian	China	Nasopharyngeal carcinoma	PB	529	577	175/96/20	123/79/8	135/73/12	209/109/21	NA	NA	NA	NA	PCR-RFLP
14	Wu	2010	Asian	Taiwan	Prostate cancer	PB	218	436	116/61^a^	164/172^a^	23/18^a^	57/43^a^	NA	NA	NA	NA	PCR
15	Liu	2009	Asian	Taiwan	Lung cancer	HB	358	716	172/98/23	282/232/49	33/26/6	80/59/14	NA	NA	NA	NA	PCR-RFLP
16	Rouissi	2009	Caucasian	Tunisia	Bladder cancer	PB	185	191	50/44/8	30/31/5	14/17/1	33/32/9	NA	NA	NA	NA	PCR-RFLP
17	Platek	2009	Mixed	America	Breast cancer	PB	1,063	1,890	NA	NA	NA	NA	351/365/98	641/726/181	78/81/21	147/129/20	PCR
18	Lin	2008	Asian	Taiwan	Colorectal cancer	HB	362	362	65/22/4	41/32/11	167/86/18	144/102/32	29/13/2	22/23/6	203/95/20	163/111/37	PCR
19	Ni	2008	Asian	China	Laryngeal cancer	PB	207	400	39/132^a^	82/60^a^	9/27^a^	70/188^a^	NA	NA	NA	NA	PCR-RFLP
20	Suzuki	2008	Asian	Japan	Pancreatic caner	HB	157	785	NA	NA	NA	NA	37/49/12	163/204/68	13/27/6	113/143/50	PCR
21	He	2007	Asian	China	Gastric cancer	PB	467	540	34/98/74	56/84/69	31/95/109	52/123/94	NA	NA	NA	NA	PCR-RFLP
22	He	2007	Asian	China	Esophageal cancer	PB	584	540	31/129/116	56/84/69	39/128/125	52/123/94	NA	NA	NA	NA	PCR-RFLP
23	Mu	2007	Asian	China	Liver cancer	HB	204	415	26/75^a^	69/119^a^	23/58^a^	66/136^a^	20/51^a^	82/44^a^	29/82^a^	171/91^a^	PCR-RFLP
24	Suzuki	2007	Asian	Japan	Head and neck cancer	HB	237	711	96/18^b^	201/41^b^	48/8^b^	211/45^b^	142/16^b^	354/74^b^	45/11^b^	193/42^b^	PCR
25	Wang	2007	Asian	China	Gastric cancer	PB	467	540	34/98/74	56/84/69	31/95/109	52/123/94	NA	NA	NA	NA	PCR-RFLP
26	Wang	2007	Asian	China	Esophageal cancer	PB	584	540	31/129/116	56/84/69	39/128/125	52/123/94	NA	NA	NA	NA	PCR-RFLP
27	Suzuki	2007	Asian	Japan	Lung cancer	HB	515	1,030	NA	NA	NA	NA	39/49/17	236/298/118	15/23/3	143/176/59	PCR
28	Song	2006	Asian	China	Rectal cancer	PB	113	414	16/23^a^	75/93^a^	9/7^a^	17/29^a^	14/16^a^	58/67^a^	30/50^a^	115/167^a^	PCR-RFLP
29	Song	2006	Asian	China	Colon cancer	PB	93	414	14/25^a^	75/93^a^	6/3^a^	17/29^a^	14/13^a^	58/67^a^	17/48^a^	115/167^a^	PCR-RFLP
30	Shi	2005	Caucasian	America	Lung cancer	HB	1,051	1,141	403/385/87	408/438/111	80/83/13	90/81/14	275/282/62	334/341/72	163/152/28	134/152/40	PCR
31	Wang	2005	Asian	China	Pancreatic caner	HB	163	337	15/67^a^	83/76^a^	16/65^a^	59/119^a^	10/41^a^	44/44^a^	21/91^a^	91/158^a^	PCR-RFLP
32	Yang	2005	Asian	Japan	Esophageal cancer	HB	165	495	35/42/13	74/103/36	28/40/7	112/124/44	39/48/7	24/40/17	24/34/13	162/187/63	PCR
33	Jiang	2005	Asian	China	Colon cancer	PB	53	343	NA	NA	NA	NA	10/7^a^	46/63^a^	9/27^a^	86/144^a^	PCR-RFLP
34	Jiang	2005	Asian	China	Rectal cancer	PB	73	343	NA	NA	NA	NA	11/7^a^	46/63^a^	21/33^a^	86/144^a^	PCR-RFLP
35	Bi	2005	Asian	China	Gastric cancer	HB	155	188	NA	NA	NA	NA	44/60^a^	49/52^a^	49/35^a^	31/23^a^	PCR-RFLP
36	Zhou	2005	Asian	China	Colorectal cancer	HB	478	838	84/112^a^	141/165^a^	45/32^a^	67/88^a^	69/83^a^	98/106^a^	60/61^a^	110/147^a^	PCR-RFLP
37	Lin	2004	Caucasian	America	Bladder cancer	PB	410	410	132/175^a^	103/115^a^	44/59^a^	93/93^a^	NA	NA	NA	NA	PCR-RFLP
38	Mu	2004	Asian	China	Gastric cancer	PB	206	415	28/76^a^	69/119^a^	21/64^a^	66/136^a^	98/42^a^	171/82^a^	35/14^a^	91/44^a^	PCR-RFLP
39	Wu	2004	Asian	China	Gastric cancer	HB	89	223	12/56^a^	34/85^a^	4/17^a^	31/67^a^	NA	NA	NA	NA	PCR
40	Zhang	2004	Caucasian	German	Esophageal cancer	HB	241	256	54/46/22	58/59/20	6/6/2	39/44/11	NA	NA	NA	NA	PCR
41	Zhang	2004	Asian	China	Esophageal cancer	HB	189	141	8/54/40	13/28/31	8/39/40	12/26/31	NA	NA	NA	NA	PCR
42	Jeng	2003	Asian	China	Lung cancer	PB	59	232	16/15^a^	41/43^a^	18/8^a^	74/56^a^	NA	NA	NA	NA	PCR-RFLP
43	Gao	2002	Asian	China	Gastric cancer	PB	107	200	19/62^a^	33/78^a^	3/22^a^	29/56^a^	9/29^a^	16/33^a^	13/52^a^	47/103^a^	PCR-RFLP
44	Matsuo	2002	Asian	Japan	Colorectal cancer	HB	72	242	31/44^a^	52/49^a^	30/75^a^	65/37^a^	NA	NA	NA	NA	PCR

HB: Hospital-based; PB: Population-based; NA: not available. ^a^ dominant model (TT + CT/CC); ^b^ recessive model (CC/CT + TT).

**Table 2:** Characteristics of the eligible studies for A1298C polymorphism among smokers, non-smokers, drinkers, and non-drinkers

No.	Author	Year	Ethnicity	Country	Cancer type	Source of control	Sample size of case	Sample size of control	Smoking		Non-smoking		Drinking		Non-drinking		
									Case: AA/AC/CC	Control: AA/AC/CC	Case: AA/AC/CC	Control: AA/AC/CC	Case: AA/AC/CC	Control: AA/AC/CC	Case: AA/AC/CC	Control: AA/AC/CC	Genotyping Method
1	Nakao	2016	Asian	Japan	Pancreatic cancer	HB	360	400	145/62/8	142/49/7	95/45/5	143/53/6	152/68/6	184/62/7	88/39/7	101/40/6	PCR-RFLP
2	Peres	2016	Mixed	Brazil	Liver cancer	HB	71	356	19/20^a^	85/72^a^	13/19^a^	119/80^a^	21/22^a^	91/75^a^	11/17^a^	113/77^a^	PCR
3	Svensson	2016	Asian	Japan	Colorectal cancer	PB	356	709	NA	NA	NA	NA	103/37/8	167/88/6	127/61/12	266/137/19	SNP
4	Al-Motassem	2015	Caucasian	Jordan	Lung cancer	PB	98	89	20/37/18	11/10/1	8/6/6	21/34/10	NA	NA	NA	NA	PCR
5	Galbiatti	2011	Mixed	Brazil	Head and neck cancer	HB	322	531	120/143^a^	128/87^a^	28/32^a^	188/128^a^	105/118^a^	160/102^a^	42/58^a^	156/113^a^	PCR-RFLP
6	Cao	2010	Asian	China	Nasopharyngeal carcinoma	PB	529	577	156/108/14	127/73/8	82/113/11	197/120/14	NA	NA	NA	NA	PCR-RFLP
7	Arslan	2010	Asian	China	Lung cancer	PB	64	61	27/23/4	26/19/3	2/6/2	2/10/1	NA	NA	NA	NA	PCR-RFLP
8	Naghibalhossaini	2010	Mixed	Iran	Colorectal cancer	PB	175	231	21/23/7	9/12/3	17/29/5	17/23/10	NA	NA	NA	NA	PCR-RFLP
9	Promthet	2010	Asian	Thailand	Colon cancer	HB	130	130	NA	NA	NA	NA	17/38/2	19/25/2	26/46/1	35/46/3	PCR-RFLP
10	Rouissi	2009	Caucasian	Tunisia	Bladder cancer	PB	185	191	40/22/4	50/44/8	46/23/5	12/19/1	NA	NA	NA	NA	PCR-RFLP
11	Zhou	2008	Asian	China	Colorectal cancer	HB	478	838	127/67^a^	180/126^a^	44/33^a^	94/61^a^	102/49^a^	128/86^a^	70/51^a^	156/101^a^	multiple PCR
12	Suzuki	2007	Asian	Japan	Lung cancer	HB	515	1,030	NA	NA	NA	NA	65/35/4	420/199/25	29/11/1	232/123/20	PCR
13	Suzuki	2008	Asian	Japan	Head and neck cancer	HB	237	711	111/1^b^	229/11^b^	55/1^b^	242/12^b^	151/6^b^	409/17^b^	55/1^b^	223/11^b^	PCR
14	Song	2006	Asian	China	Colon cancer	PB	93	414	104/64^a^	26/12^a^	31/15^a^	4/5^a^	74/51^a^	15/12^a^	189/95^a^	42/19^a^	PCR-RFLP
15	Song	2006	Asian	China	Rectal cancer	PB	114	414	14/2^a^	26/12^a^	27/11^a^	4/5^a^	56/22^a^	15/12^a^	19/11^a^	42/19^a^	PCR-RFLP
16	Shi	2005	Caucasian	America	Lung cancer	HB	1,051	1,141	692/389/94	473/416/77	88/73/15	91/80/14	285/268/66	321/279/57	150/162/31	173/124/29	PCR
17	Jiang	2005	Asian	China	Colon cancer	PB	53	343	NA	NA	NA	NA	9/8^a^	69/38^a^	27/9^a^	157/71^a^	PCR-RFLP
18	Jiang	2005	Asian	China	Rectal cancer	PB	73	343	NA	NA	NA	NA	12/6^a^	69/38^a^	45/8^a^	157/71^a^	PCR-RFLP
19	Lin	2004	Caucasian	America	Bladder cancer	PB	410	410	142/165^a^	110/114^a^	50/53^a^	79/106^a^	NA	NA	NA	NA	PCR-RFLP
20	Matsuo	2002	Asian	Japan	Colorectal cancer	HB	72	242	48/26^a^	70/31^a^	46/21^a^	87/53^a^	NA	NA	NA	NA	PCR

HB: Hospital-based; PB: Population-based; NA: not available. ^a^ dominant model (CC + AC/AA); ^b^ recessive model (AA/CC + AC).

In the final analysis, 36 articles investigated the relationship between C677T polymorphism and cancers among smokers, while 27 articles focused on the relationship between C677T polymorphism and cancers among drinkers. As for the A1298C gene, 19 articles were relevant, with 16 of them exploring the association between A1298C polymorphism and cancers among smokers and 12 articles examining the association between A1298C polymorphism and cancers among drinkers.

#### 3.1.1 C677T polymorphism among smokers and drinkers

Among the 36 smoking-related literatures, there were 5 studies focused on lung cancer, 6 studies for gastric cancer, 6 studies for esophageal cancer, 3 studies for colorectal cancer, 2 studies for pancreatic cancer, 2 studies for liver cancer, and 2 studies for bladder cancer. The remaining types of cancers, such as breast cancer and thyroid cancer, did not have enough studies for subgroup analysis and were represented by less than one article each. In terms of control group source, 17 studies were hospital-based, and 19 studies were population-based. Regarding ethnicity, 28 studies included Asian population, 4 studies included Caucasian population, and 4 studies included mixed population.

Among the 27 alcohol-related literature, there were 4 studies focused on lung cancer, 3 studies on pancreatic cancer, 3 studies on colorectal cancer, 4 studies on gastric cancer, 2 studies on liver cancer, 2 studies on esophageal cancer, 2 studies on colon cancer, and 2 studies on rectal cancer. In terms of control group source, 16 studies were hospital-based and 11 studies were population-based. Regarding ethnicity, 20 studies included Asian population, 2 studies included Caucasian population, and 5 studies included mixed population. Table 1 provides a summary of the characteristics of the included studies for the C677T polymorphism among smokers and drinkers.

#### 3.1.2 A1298C polymorphism among smokers and drinkers

Among the 15 smoking-related studies, there were 3 studies focused on lung cancer, 3 studies on colorectal cancer, and 2 studies on bladder cancer. For other types of cancer such as colon cancer, head and neck cancer, and liver cancer, there were fewer than 2 studies conducted for each, thus subgroup analysis was not performed. In terms of control group source, 7 studies were hospital-based and 8 studies were population-based. Regarding ethnicity, 8 studies included Asian population, 4 studies included Caucasian population, and 3 studies included mixed population.

For the 13 alcohol-related studies, there were 3 studies on colon cancer, 2 studies on colorectal cancer, 2 studies on rectal cancer, and others. Concerning the control group source, 7 studies were hospital-based and 6 studies were population-based. Regarding ethnicity, 10 studies included Asian population and 2 studies included mixed population. Table 2 provides the characteristics of the included studies for the A1298C polymorphism among smokers and drinkers.

### 3.2 Quantitative synthesis

#### 3.2.1 Relationship between C677T polymorphism and cancer among smokers, non-smokers, drinkers, and non-drinkers

The analysis of the included articles revealed that the C677T polymorphism significantly increased overall cancer susceptibility among smokers in the dominant model (*I* ^2^ = 83.20%, CT + TT vs CC, OR [95% CI] = 1.225 [1.009–1.487], *p* = 0.041), However, no significant association was observed among non-smokers. In subgroup analyses, Asian smokers were to have that elevated risk of cancer with 1.292-fold in the dominant model, whereas Asian non-smokers were not. No association was observed among Caucasian smokers or non-smokers. Liver cancer risk was elevated among smokers by 1.564-fold, compared to non-smokers. Furthermore, smokers had a 1.564-fold increased risk of liver cancer compared to non-smokers. The risk of esophageal cancer was significantly increased in both smokers and non-smokers. However, no significant difference was found between smokers and non-smokers in terms of the association between the C677T polymorphism and other types of cancer such as lung cancer and gastric cancer. The detailed results are presented in Table 3.

**Table 3:** Integral analysis of the association between C677T polymorphism and cancer risk among smoking population

Comparative model	No.	*Z*	*p*	OR (95% CI)	Heterogeneity			*Z*	Begg’s Test	*t*	Egger’s test	FPRP *p*-value^a^	FPRP statistical power^b^	FPRP prior probability					BFDP prior probability		
					Heterogeneity chi-squared	*p*	*I* ^2^							0.25	0.10	0.01	0.001	0.0001	0.01	0.001	0.00001
**Smoker**																					
**TT/CC**																					
**Overall**	17	0.79	0.432	1.135 (0.827–1.558)	64.71	0.00	75.30%	0.12	0.902	0.29	0.779	0.433	0.958	0.576	0.803	0.978	0.998	1.000	0.996	1.000	1.000
**Ethnicity**																					
Caucasian	3	1.13	0.259	0.852 (0.645–1.125)	1.05	0.59	0.00%	0.00	1.000	1.14	0.459	0.259	0.958	0.448	0.708	0.964	0.996	1.000	0.996	1.000	1.000
Asian	14	0.83	0.404	1.177 (0.802–1.728)	58.81	0.00	77.90%	0.77	0.443	−0.46	0.657	0.406	0.892	0.577	0.804	0.978	0.998	1.000	0.995	1.000	1.000
**Source of control**																					
HB	8	2.17	0.030	0.798 (0.651–0.978)	10.48	0.16	33.20%	0.37	0.711	−0.25	0.814	0.030	0.958	**0.085**	0.218	0.754	0.969	0.997	0.982	0.998	1.000
PB	9	1.95	0.051	1.582 (0.998–2.508）	34.21	0.00	76.60%	0.31	0.754	0.13	0.903	0.051	0.410	0.272	0.528	0.925	0.992	0.999	0.981	0.998	1.000
**Cancer types**																					
Lung cancer	4	1.45	0.148	0.833 (0.650–1.067)	2.38	0.50	0.00%	1.70	0.089	2.65	0.118	0.148	0.961	0.316	0.581	0.938	0.994	0.999	0.994	0.999	1.000
Gastric cancer	3	0.54	0.591	1.218 (0.594–2.498)	8.23	0.02	75.70%	0.52	0.602	.	.	0.590	0.715	0.712	0.881	0.988	0.999	1.000	0.994	0.999	1.000
Esophageal cancer	5	2.17	0.030	**1.834 (1.061**–**3.171)**	13.18	0.01	69.70%	0.98	0.327	−1.37	0.264	0.030	0.236	0.276	0.533	0.926	0.992	0.999	0.971	0.997	1.000
**CT/CC**																					
**Overall**	17	0.24	0.814	1.029 (0.809–1.311)	81.17	0.00	80.30%	1.36	0.174	1.45	0.167	0.817	0.999	0.710	0.880	0.988	0.999	1.000	0.998	1.000	1.000
**Ethnicity**																					
Caucasian	3	1.41	0.158	0.881 (0.739–1.050)	0.06	0.97	0.00%	0.00	1.000	−3.00	0.205	0.157	0.999	0.320	0.586	0.940	0.994	0.999	0.996	1.000	1.000
Asian	14	0.49	0.623	1.082 (0.791–1.480)	80.71	0.00	83.90%	1.20	0.228	1.82	0.093	0.622	0.980	0.656	0.851	0.984	0.998	1.000	0.997	1.000	1.000
**Source of control**																					
HB	8	2.20	0.028	0.758 (0.592–0.970)	17.44	0.02	59.90%	0.37	0.711	−0.22	0.832	0.028	0.846	**0.089**	0.227	0.764	0.970	0.997	0.978	0.998	1.000
PB	9	1.45	0.148	1.339 (0.902–1.988)	51.14	0.00	84.40%	1.56	0.118	2.17	0.066	0.148	0.713	0.383	0.651	0.953	0.995	1.000	0.991	0.999	1.000
**Cancer types**																					
Lung cancer	4	1.95	0.051	0.862 (0.743–1.001)	3.39	0.34	11.60%	0.34	0.734	0.42	0.717	0.052	1.000	**0.134**	0.317	0.836	0.981	0.998	0.991	0.999	1.000
Gastric cancer	3	0.35	0.726	1.184 (0.459–3.053)	20.07	0.00	90.00%	0.52	0.602			0.727	0.688	0.760	0.905	0.991	0.999	1.000	0.993	0.999	1.000
Esophageal cancer	5	1.76	0.079	1.714 (0.939–3.129)	21.10	0.00	81.00%	0.49	0.624	0.37	0.734	0.079	0.332	0.417	0.683	0.959	0.996	1.000	0.984	0.998	1.000
**CT + TT/CC**																					
**Overall**	33	2.05	0.041	**1.225 (1.009**–**1.487)**	189.95	0.00	83.20%	1.78	0.075	2.78	**0.009**	0.040	0.980	**0.109**	0.269	0.802	0.976	0.998	0.987	0.999	1.000
**Ethnicity**																					
Caucasian	4	0.99	0.323	0.927 (0.798–1.077)	2.43	0.49	0.00%	−0.34	1.000	0.55	0.639	0.322	1.000	0.491	0.743	0.970	0.997	1.000	0.998	1.000	1.000
Asian	27	2.02	0.044	**1.292 (1.007**–**1.658)**	182.94	0.00	85.80%	1.38	0.169	2.71	**0.012**	0.044	0.880	**0.131**	0.311	0.832	0.980	0.998	0.985	0.998	1.000
Mix	2	0.18	0.859	1.030 (0.742–1.429)	0.75	0.39	0.00%	0.00	1.000	–	–	0.860	0.988	0.723	0.887	0.989	0.999	1.000	0.997	1.000	1.000
**Source of control**																					
HB	16	0.74	0.460	1.097 (0.858–1.402)	70.04	0.00	78.60%	2.03	**0.043**	1.81	0.092	0.460	0.994	0.581	0.806	0.979	0.998	1.000	0.997	1.000	1.000
PB	17	1.91	0.056	1.347 (0.992–1.828)	112.67	0.00	85.80%	0.78	0.434	1.93	0.072	0.056	0.755	**0.182**	0.400	0.880	0.987	0.999	0.985	0.999	1.000
**Cancer types**																					
Lung cancer	5	2.17	0.030	0.857 (0.745–0.985)	3.67	0.45	0.00%	−0.24	1.000	0.77	0.495	0.030	1.000	**0.082**	0.211	0.747	0.968	0.997	0.987	0.999	1.000
Gastric cancer	6	1.23	0.220	1.347 (0.837–2.169)	22.53	0.75	77.80%	0.56	0.573	0.26	0.807	0.220	0.671	0.496	0.747	0.970	0.997	1.000	0.992	0.999	1.000
Esophageal cancer	6	2.25	0.024	**1.755 (1.076**–**2.863)**	22.63	0.00	78.80%	0.56	0.573	0.26	0.806	0.024	0.265	0.216	0.452	0.901	0.989	0.999	0.967	0.997	1.000
Colorectal cancer	3	0.33	0.739	0.884 (0.430–1.820)	11.40	0.00	82.40%	0.00	1.000	−0.50	0.707	0.738	0.778	0.740	0.895	0.989	0.999	1.000	0.994	0.999	1.000
Pancreatic cancer	2	0.69	0.489	1.901 (0.309–11.712)	22.88	0.00	95.60%	0.00	1.000			0.489	0.399	0.786	0.917	0.992	0.999	1.000	0.991	0.999	1.000
Liver cancer	2	2.02	0.043	**1.564 (1.014**–**2.413)**	0.18	0.68	0.00%	0.00	1.000			0.043	0.425	0.234	0.478	0.910	0.990	0.999	0.979	0.998	1.000
Bladder cancer	2	0.62	0.536	1.101 (0.812–1.492)	0.75	0.39	0.00%	0.00	1.000			0.535	0.977	0.622	0.831	0.982	0.998	1.000	0.997	1.000	1.000
**TT/CT + CC**																					
**Overall**	20	0.77	0.444	1.036 (0.946–1.136)	25.72	0.14	26.10%	0.29	0.770	−0.11	0.913	0.452	1.000	0.575	0.803	0.978	0.998	1.000	0.999	1.000	1.000
**Ethnicity**																					
Caucasian	3	0.70	0.483	0.920 (0.729–1.161)	1.40	0.50	0.00%	0.00	1.000	1.00	0.501	0.482	0.997	0.592	0.813	0.980	0.998	1.000	0.997	1.000	1.000
Asian	15	0.94	0.345	1.049 (0.949–1.160)	19.06	0.16	26.50%	1.29	0.198	−1.59	0.135	0.351	1.000	0.513	0.760	0.972	0.997	1.000	0.999	1.000	1.000
**Source of control**																					
HB	9	1.45	0.146	0.895 (0.770–1.040)	5.03	0.76	0.00%	0.73	0.466	−0.90	0.400	0.148	1.000	0.307	0.571	0.936	0.993	0.999	0.996	1.000	1.000
PB	11	2.37	0.018	**1.150 (1.024**–**1.291)**	13.99	0.17	28.50%	0.62	0.533	0.84	0.423	0.018	1.000	**0.051**	**0.138**	0.639	0.947	0.994	0.983	0.998	1.000
**Cancer types**																					
Lung cancer	4	0.98	0.329	0.899 (0.726–1.113)	1.88	0.60	0.00%	1.70	0.089	2.97	0.097	0.328	0.997	0.497	0.748	0.970	0.997	1.000	0.997	1.000	1.000
Gastric cancer	3	0.57	0.567	1.054 (0.880–1.264)	0.79	0.67	0.00%	0.52	0.602			0.570	1.000	0.631	0.837	0.983	0.998	1.000	0.998	1.000	1.000
Esophageal cancer	5	2.22	0.027	**1.175 (1.019**–**1.356)**	3.98	0.41	0.00%	0.98	0.327	−1.48	0.235	0.027	1.000	**0.076**	**0.198**	0.731	0.965	0.996	0.986	0.999	1.000
**Non-smoker**																					
**TT/CC**																					
**Overall**	17	0.12	0.906	1.017 (0.764–1.355)	42.31	0.00	62.20%	0.45	0.650	−0.67	0.511	0.908	0.996	0.732	0.891	0.989	0.999	1.000	0.997	1.000	1.000
**Ethnicity**																					
Caucasian	3	0.40	0.689	0.870 (0.441–1.716)	1.50	0.47	0.00%	1.04	0.296	−0.87	0.543	0.688	0.779	0.726	0.888	0.989	0.999	1.000	0.994	0.999	1.000
Asian	14	0.21	0.833	1.034 (0.756–1.414)	40.67	0.00	68.00%	0.11	0.913	−0.41	0.690	0.834	0.990	0.717	0.883	0.988	0.999	1.000	0.997	1.000	1.000
**Source of control**																					
HB	8	2.07	0.039	0.759 (0.585–0.986)	8.40	0.30	16.70%	0.37	0.711	1.64	0.152	0.039	0.834	**0.123**	0.295	0.822	0.979	0.998	0.983	0.998	1.000
PB	9	0.91	0.361	1.204 (0.809–1.792)	24.46	0.00	67.30%	0.10	0.917	−0.87	0.413	0.360	0.861	0.557	0.790	0.976	0.998	1.000	0.995	1.000	1.000
**Cancer types**																					
Lung cancer	4	1.81	0.070	0.709 (0.488–1.028)	2.13	0.55	0.00%	−0.34	1.000	1.27	0.331	0.070	0.627	0.250	0.500	0.917	0.991	0.999	0.986	0.999	1.000
Gastric cancer	3	0.62	0.535	1.290 (0.577–2.884)	14.15	0.00	85.90%	0.52	0.602			0.535	0.643	0.714	0.882	0.988	0.999	1.000	0.993	0.999	1.000
Esophageal cancer	5	2.87	0.004	**1.550 (1.149**–**2.092)**	4.62	0.33	13.40%	0.98	0.327	−0.94	0.418	0.004	0.415	**0.029**	**0.083**	0.499	0.909	0.990	0.892	0.988	1.000
**CT/CC**																					
**Overall**	17	0.25	0.804	0.985 (0.878–1.106)	19.47	0.25	17.80%	0.95	0.343	1.37	0.191	0.798	1.000	0.705	0.878	0.988	0.999	1.000	0.999	1.000	1.000
**Ethnicity**																					
Caucasian	3	0.71	0.476	1.142 (0.792–1.647)	0.21	0.90	0.00%	0.00	1.000	−0.56	0.674	0.477	0.928	0.607	0.822	0.981	0.998	1.000	0.996	1.000	1.000
Asian	14	0.50	0.617	0.969 (0.858–1.095)	18.55	0.14	29.90%	1.20	0.228	1.41	0.184	0.614	1.000	0.648	0.847	0.984	0.998	1.000	0.999	1.000	1.000
**Source of control**																					
HB	8	0.88	0.377	0.923 (0.773–1.103)	8.89	0.27	21.20%	1.61	0.108	1.89	0.108	0.378	1.000	0.531	0.773	0.974	0.997	1.000	0.998	1.000	1.000
PB	9	0.43	0.667	1.034 (0.888–1.204)	9.67	0.29	17.30%	−0.10	1.000	0.52	0.620	0.667	1.000	0.667	0.857	0.985	0.999	1.000	0.998	1.000	1.000
**Cancer types**																					
Lung cancer	4	0.66	0.512	0.931 (0.752–1.153)	2.32	0.51	0.00%	−0.34	1.000	−0.11	0.922	0.512	0.999	0.606	0.822	0.981	0.998	1.000	0.998	1.000	1.000
Gastric cancer	3	0.14	0.886	1.031 (0.679–1.565)	4.70	0.10	57.40%	0.52	0.602			0.886	0.961	0.734	0.892	0.989	0.999	1.000	0.996	1.000	1.000
Esophageal cancer	5	2.32	0.020	**1.379 (1.051**–**1.808)**	1.45	0.84	0.00%	0.49	0.624	0.07	0.945	0.020	0.729	**0.076**	**0.199**	0.732	0.965	0.996	0.970	0.997	1.000
**CT + TT/CC**																					
**Overall**	33	1.37	0.171	1.123 (0.951–1.325)	88.16	0.00	63.70%	0.36	0.722	1.19	0.242	0.169	1.000	0.337	0.604	0.944	0.994	0.999	0.996	1.000	1.000
**Ethnicity**																					
Caucasian	4	1.12	0,262	1.177 (0.885–1.565)	0.54	0.91	0.00%	1.02	0.308	−1.07	0.397	0.262	0.952	0.452	0.713	0.965	0.996	1.000	0.995	1.000	1.000
Asian	27	1.34	0.180	1.145 (0.940–1.395)	86.15	0.00	69.80%	0.79	0.428	1.32	0.200	0.179	0.996	0.350	0.618	0.947	0.994	0.999	0.996	1.000	1.000
Mix	2	0.72	0.469	0.848 (0.544–1.324)	0.00	0.99	0.00%	0.00	1.000			0.468	0.855	0.622	0.831	0.982	0.998	1.000	0.995	1.000	1.000
**Source of control**																					
HB	16	1.06	0.290	1.158 (0.882–1.521)	56.09	0.00	73.30%	2.03	**0.043**	1.97	0.069	0.292	0.969	0.475	0.730	0.968	0.997	1.000	0.996	1.000	1.000
PB	17	1.26	0.209	1.084 (0.956–1.230)	31.90	0.01	49.80%	0.95	0.343	0.04	0.972	0.211	1.000	0.387	0.655	0.954	0.995	1.000	0.998	1.000	1.000
**Cancer types**																					
Lung cancer	5	1.37	0.172	0.871 (0.714–1.062)	3.76	0.44	0.00%	−2.24	1.000	−0.26	0.808	0.172	0.996	0.341	0.609	0.945	0.994	0.999	0.996	1.000	1.000
Gastric cancer	6	1.51	0.130	1.410 (0.904–2.199)	16.27	0.01	69.30%	0.94	0.348	2.12	0.101	0.130	0.608	0.390	0.658	0.955	0.995	1.000	0.990	0.999	1.000
Esophageal cancer	6	3.62	0.000	**1.540 (1.219**–**1.945)**	3.98	0.55	0.00%	−0.19	1.000	−0.10	0.922	0.000	0.413	**0.002**	**0.006**	**0.065**	0.412	0.875	**0.470**	0.900	0.999
Colorectal cancer	3	0.24	0.811	1.153 (0.360–3.688)	34.37	0.00	94.20%	1.04	0.296	0.69	0.614	0.810	0.671	0.784	0.916	0.992	0.999	1.000	0.993	0.999	1.000
Pancreatic cancer	2	0.70	0.484	1.317 (0.609–2.845)	3.95	0.05	74.70%	0.00	1.000			0.483	0.630	0.697	0.874	0.987	0.999	1.000	0.993	0.999	1.000
Liver cancer	2	0.30	0.762	1.072 (0.684–1.680)	0.60	0.44	0.00%	0.00	1.000			0.762	0.929	0.711	0.881	0.988	0.999	1.000	0.996	1.000	1.000
Bladder cancer	2	1.07	0.286	1.256 (0.826–1.910)	0.28	0.60	0.00%	0.00	1.000			0.287	0.797	0.519	0.764	0.973	0.997	1.000	0.994	0.999	1.000
**TT/CT + CC**																					
**Overall**	20	1.28	0.202	1.084 (0.958–1.226)	34.28	0.02	44.60%	0.23	0.820	−2.12	**0.048**	0.199	1.000	0.374	0.642	0.952	0.995	0.999	0.997	1.000	1.000
**Ethnicity**																					
Caucasian	3	0.61	0.543	0.816 (0.425–1.568)	1.82	0.40	0.00%	1.04	0.296	−0.74	0.596	0.542	0.728	0.691	0.870	0.987	0.999	1.000	0.994	0.999	1.000
Asian	15	0.01	0.994	1.001 (0.808–1.240)	31.77	0.00	55.90%	0.69	0.488	−2.15	0.051	0.993	1.000	0.749	0.899	0.990	0.999	1.000	0.998	1.000	1.000
**Source of control**																					
HB	9	2.01	0.045	0.795 (0.636–0.994)	4.90	0.77	0.00%	−0.10	1.000	0.51	0.628	0.044	0.939	**0.124**	0.297	0.823	0.979	0.998	0.986	0.999	1.000
PB	11	2.93	0.003	**1.250 (1.077**–**1.451)**	18.41	0.05	45.70%	1.25	0.213	−1.52	0.162	0.003	0.992	**0.010**	**0.030**	0.251	0.772	0.971	0.915	0.991	1.000
**Cancer types**																					
Lung cancer	4	1.66	0.096	0.738 (0.516–1.056)	1.58	0.66	0.00%	−0.34	1.000	2.22	0.156	0.097	0.711	0.289	0.550	0.931	0.993	0.999	0.989	0.999	1.000
Gastric cancer	3	0.85	0.394	1.242 (0.755–2.043)	9.57	0.01	79.10%	0.52	0.602			0.393	0.771	0.605	0.821	0.981	0.998	1.000	0.994	0.999	1.000
Esophageal cancer	5	2.07	0.038	**1.253 (1.012**–**1.551)**	4.70	0.32	15.00%	0.98	0.327	−1.50	0.231	0.038	0.951	**0.108**	0.266	0.799	0.976	0.998	0.985	0.998	1.000

Bold values indicates statistically significant OR values, values with FPRP less than 0.2, and values with BFDP less than 0.8.

The analysis revealed that the overall risk of cancer was higher among non-drinkers in the dominant model (*I* ^2^ = 80.50%, CT + TT vs CC, OR [95% CI] = 1.248 [1.001–1.557], *p* = 0.049), compared with drinkers. Subgroup analyses found that there was no significant difference between drinkers and non-drinkers in Asians, as well as a mixed population. Additionally, non-drinkers had a 1.544-fold increased risk of esophageal cancer and a 1.877-fold increased risk of colon cancer compared to drinkers. The detailed results are presented in Table 4.

**Table 4:** Integral analysis of the association between C677T polymorphism and cancer risk among drinking population

Comparative model	No.	*Z*	*p*	OR (95% CI)	Heterogeneity			*Z*	Begg’s Test	*t*	Egger’s test	FPRP *p*-value^a^	FPRP statistical power^b^	FPRP prior probability					BFDP prior probability		
					Heterogeneity chi-squared	*p*	*I* ^2^							0.25	0.10	0.01	0.001	0.0001	0.01	0.001	0.000010
**Drinker**																					
**TT/CC**	11	1.60	0.109	0.881 (0.754–1.029)	14.68	0.144	31.90%	2.49	**0.013**	−4.27	**0.002**	0.110	1.000	0.248	0.497	0.916	0.991	0.999	0.995	1.000	1.000
**Overall**																					
**Ethnicity**																					
Asian	8	3.07	0.002	0.667 (0.515–0.864)	7.64	0.366	8.40%	1.61	0.108	−2.62	**0.040**	0.002	0.502	**0.013**	**0.037**	0.299	0.811	0.977	0.834	0.981	1.000
**Source of control**																					
HB	8	1.74	0.083	0.837 (0.685–1.023)	13.69	0.057	48.90%	2.10	**0.035**	−4.79	**0.003**	0.082	0.987	**0.200**	0.429	0.892	0.988	0.999	0.992	0.999	1.000
PB	3	0.41	0.682	0.950 (0.744–1.213)	0.47	0.791	0.00%	1.04	0.296	−14.98	**0.042**	0.681	0.998	0.672	0.860	0.985	0.999	1.000	0.998	1.000	1.000
**Cancer types**																					
Lung cancer	4	0.17	0.863	1.021 (0.802–1.301)	1.00	0.802	0.00%	1.02	0.308	−3.51	0.072	0.867	0.999	0.722	0.886	0.988	0.999	1.000	0.998	1.000	1.000
Pancreatic cancer	2	1.58	0.115	0.715 (0.472–1.085)	0.08	0.775	0.00%	0.00	1.000			0.115	0.629	0.354	0.622	0.948	0.995	0.999	0.989	0.999	1.000
Colorectal cancer	2	1.01	0.311	0.755 (0.438–1.301)	1.86	0.173	46.20%	0.00	1.000			0.311	0.673	0.581	0.806	0.979	0.998	1.000	0.993	0.999	1.000
**CT/CC**																					
**Overall**	11	2.51	0.012	0.799 (0.670–0.952)	21.56	0.017	53.60%	1.40	0.161	−2.04	0.071	0.012	0.979	**0.036**	**0.100**	0.550	0.925	0.992	0.967	0.997	1.000
**Ethnicity**																					
Asian	8	3.88	0.000	0.697 (0.581–0.836)	13.84	0.054	49.40%	0.12	0.902	−0.43	0.682	0.000	0.684	**0.000**	**0.001**	**0.014**	**0.127**	0.594	**0.277**	**0.794**	0.997
**Source of control**																					
HB	8	1.87	0.061	0.879 (0.768–1.006)	12.90	0.074	45.80%	1.36	0.174	−2.44	0.051	0.061	1.000	**0.155**	0.355	0.858	0.984	0.998	0.993	0.999	1.000
PB	3	1.29	0.196	0.733 (0.457–1.174)	8.39	0.015	76.20%	0.00	1.000	−0.64	0.636	0.196	0.653	0.474	0.730	0.967	0.997	1.000	0.992	0.999	1.000
**Cancer types**																					
Lung cancer	4	0.30	0.764	0.976 (0.833–1.144)	0.18	0.980	0.00%	0.34	0.734	−0.64	0.585	0.764	1.000	0.696	0.873	0.987	0.999	1.000	0.998	1.000	1.000
Pancreatic cancer	2	1.11	0.269	0.840 (0.616–1.145)	1.61	0.204	38.10%					0.270	0.928	0.466	0.724	0.966	0.997	1.000	0.995	1.000	1.000
Colorectal cancer	2	4.00	0.000	0.460 (0.314–0.673)	0.03	0.862	0.00%					0.000	0.028	**0.007**	**0.020**	**0.183**	0.694	0.958	**0.193**	**0.707**	0.996
**CT + TT/CC**																					
**Overall**	24	0.41	0.681	0.965 (0.812–1.146)	74.01	0.000	68.90%	0.12	0.901	0.18	0.862	0.685	1.000	0.673	0.860	0.985	0.999	1.000	0.998	1.000	1.000
**Ethnicity**																					
Asian	19	0.37	0.714	0.951 (0.727–1.244)	72.67	0.000	75.20%	0.00	1.000	0.42	0.681	0.714	0.995	0.683	0.866	0.986	0.999	1.000	0.997	1.000	1.000
Mix	3	0.61	0.541	0.954 (0.820–1.110)	0.81	0.667	0.00%	1.04	0.296	2.56	0.237	0.542	1.000	0.619	0.830	0.982	0.998	1.000	0.998	1.000	1.000
**Source of control**																					
HB	15	0.49	0.627	1.061 (0.835–1.349)	60.21	0.000	76.70%	0.68	0.488	0.58	0.573	0.629	0.998	0.654	0.850	0.984	0.998	1.000	0.998	1.000	1.000
PB	9	2.25	0.024	0.855 (0.747–0.980)	9.98	0.267	19.80%	0.73	0.466	−0.83	0.434	0.024	1.000	**0.068**	**0.180**	0.708	0.961	0.996	0.985	0.999	1.000
**Cancer types**																					
Lung cancer	4	0.18	0.858	0.986 (0.849–1.146)	0.21	0.976	0.00%	1.70	0.089	−4.41	**0.048**	0.854	1.000	0.719	0.885	0.988	0.999	1.000	0.999	1.000	1.000
Pancreatic cancer	3	0.64	0.522	1.307 (0.576–2.963)	15.05	0.001	86.70%	1.04	0.296	15.06	**0.042**	0.521	0.629	0.713	0.882	0.988	0.999	1.000	0.993	0.999	1.000
Colorectal cancer	3	1.39	0.166	0.659 (0.366–1.189)	7.99	0.018	75.00%	0.00	1.000	−0.47	0.720	0.166	0.485	0.507	0.755	0.971	0.997	1.000	0.990	0.999	1.000
Gastric cancer	4	0.45	0.654	0.881 (0.507–1.531)	10.31	0.016	70.90%	−0.34	1.000	0.38	0.740	0.653	0.839	0.700	0.875	0.987	0.999	1.000	0.995	1.000	1.000
Liver cancer	2	1.42	0.155	2.511 (0.705–8.945)	7.13	0.008	86.00%	0.00	1.000			0.155	0.213	0.686	0.868	0.986	0.999	1.000	0.988	0.999	1.000
Esophageal cancer	2	0.04	0.965	0.979 (0.384–2.496)	5.61	0.018	82.20%	0.00	1.000			0.965	0.789	0.786	0.917	0.992	0.999	1.000	0.993	0.999	1.000
Colon cancer	2	1.20	0.230	0.673 (0.352–1.285)	0.44	0.505	0.00%	0.00	1.000			0.230	0.511	0.574	0.802	0.978	0.998	1.000	0.991	0.999	1.000
Rectal cancer	2	0.95	0.344	0.741 (0.398–1.379)	1.31	0.253	23.40%	0.00	1.000			0.344	0.631	0.621	0.831	0.982	0.998	1.000	0.993	0.999	1.000
**TT/CT + CC**																					
**Overall**	14	0.77	0.443	0.948 (0.826–1.087)	21.96	0.056	40.80%	0.88	0.381	−0.69	0.505	0.444	1.000	0.571	0.800	0.978	0.998	1.000	0.998	1.000	1.000
**Ethnicity**																					
Asian	9	2.52	0.012	0.757 (0.609–0.940)	10.05	0.261	20.40%	1.77	0.076	−1.83	0.109	0.012	0.875	**0.039**	**0.108**	0.570	0.931	0.993	0.960	0.996	1.000
Mix	3	1.26	0.206	1.901 (0.702–5.142)	5.33	0.070	62.50%	0.00	1.000	4.98	0.126	0.206	0.320	0.658	0.853	0.985	0.998	1.000	0.990	0.999	1.000
**Source of control**																					
HB	9	1.78	0.075	0.852 (0.715–1.016)	12.55	0.128	36.20%	2.40	**0.016**	−3.70	**0.008**	0.075	0.997	**0.183**	0.402	0.881	0.987	0.999	0.993	0.999	1.000
PB	5	1.05	0.296	1.126 (0.902–1.405)	6.30	0.178	36.50%	1.22	0.221	1.53	0.224	0.293							0.997	1.000	1.000
**Cancer types**																					
Lung cancer	4	0.29	0.771	1.034 (0.825–1.297)	1.21	0.751	0.00%	1.02	0.308	−2.44	0.135	0.772	0.999	0.699	0.874	0.987	0.999	1.000	0.998	1.000	1.000
Pancreatic cancer	2	1.09	0.274	0.814 (0.563–1.177)	0.08	0.774	0.00%	0.00	1.000			0.274	0.327	0.538	0.777	0.975	0.997	1.000	0.995	0.999	1.000
Colorectal cancer	2	0.24	0.812	0.869 (0.271–2.782)	2.10	0.148	52.30%	0.00	1.000			0.813	0.672	0.784	0.916	0.992	0.999	1.000	0.993	0.999	1.000
Breast cancer	2	0.82	0.411	1.710 (0.476–6.148)	3.67	0.056	72.70%	0.00	1.000			0.411	0.420	0.746	0.898	0.990	0.999	1.000	0.991	0.999	1.000
**Non-drinker**																					
**TT/CC**																					
**Overall**	11	0.08	0.932	0.983 (0.670–1.444)	38.63	0.000	74.10%	0.47	0.640	0.64	0.538	0.930	0.976	0.741	0.896	0.990	0.999	1.000	0.997	1.000	1.000
**Ethnicity**																					
Asian	8	0.66	0.507	0.867 (0.568–1.322)	23.71	0.001	70.50%	−0.12	1.000	−0.03	0.975	0.507	0.889	0.631	0.837	0.983	0.998	1.000	0.996	1.000	1.000
**Source of control**																					
HB	8	0.72	0.470	0.854 (0.557–1.310)	20.67	0.004	66.10%	1.11	0.266	1.18	0.283	0.470	0.872	0.618	0.829	0.982	0.998	1.000	0.995	1.000	1.000
PB	3	0.76	0.450	1.342 (0.626–2.878)	11.68	0.003	82.90%	0.00	1.000	0.43	0.740	0.450	0.613	0.688	0.869	0.986	0.999	1.000	0.994	0.999	1.000
**Cancer types**																					
Lung cancer	4	0.35	0.725	1.161 (0.507–2.660)	16.83	0.001	82.20%	0.34	0.734	−0.17	0.880	0.724	0.728	0.749	0.900	0.990	0.999	1.000	0.994	0.999	1.000
Pancreatic cancer	2	0.58	0.564	1.176 (0.679–2.036)	0.07	0.786	0.00%					0.563	0.808	0.676	0.862	0.986	0.999	1.000	0.995	0.999	1.000
Colorectal cancer	2	3.12	0.002	0.549 (0.376–0.800)	1.08	0.300	7.00%					0.002	0.156	**0.033**	**0.094**	0.533	0.920	0.991	**0.796**	0.975	1.000
**CT/CC**																					
**Overall**	11	0.58	0.565	1.079 (0.833–1.397)	41.92	0.000	76.10%	1.09	0.276	−0.06	0.953	0.564	0.994	0.630	0.836	0.983	0.998	1.000	0.997	1.000	1.000
**Ethnicity**																					
Asian	8	0.35	0.728	1.063 (0.751–1.505)	36.81	0.000	81.00%	1.36	0.174	−0.40	0.706	0.731	0.974	0.692	0.871	0.987	0.999	1.000	0.997	1.000	1.000
**Source of control**																					
HB	8	0.03	0.974	0.910 (0.778–1.065)	13.58	0.059	48.40%	1.86	0.063	4.46	**0.004**	0.240	1.000	0.419	0.683	0.960	0.996	1.000	0.997	1.000	1.000
PB	3	0.72	0.472	1.231 (0.699–2.167)	16.98	0.000	88.20%	0.00	1.000	−1.80	0.322	0.471	0.753	0.652	0.849	0.984	0.998	1.000	0.994	0.999	1.000
**Cancer types**																					
Lung cancer	4	1.18	0.240	1.342 (0.822–2.192)	17.43	0.001	82.80%	0.34	0.734	−0.29	0.800	0.240	0.672	0.517	0.763	0.973	0.997	1.000	0.993	0.999	1.000
Pancreatic cancer	2	0.88	0.378	1.207 (0.794–1.835)	1.17	0.280	14.50%	0.00	1.000			0.379	0.845	0.573	0.801	0.978	0.998	1.000	0.995	1.000	1.000
Colorectal cancer	2	2.49	0.013	0.727 (0.566–0.935)	0.22	0.635	0.00%	0.00	1.000			0.013	0.750	**0.049**	**0.135**	0.632	0.945	0.994	0.959	0.996	1.000
**CT + TT/CC**																					
**Overall**	24	1.97	0.049	**1.248 (1.001**–**1.557)**	117.97	0.000	80.50%	1.22	0.224	0.93	0.361	0.050	0.948	**0.136**	0.320	0.838	0.981	0.998	0.987	0.999	1.000
**Ethnicity**																					
Asian	19	1.81	0.071	1.286 (0.979–1.689)	106.47	0.000	83.10%	1.33	0.184	0.69	0.502	0.071	0.866	**0.196**	0.423	0.890	0.988	0.999	0.989	0.999	1.000
Mix	3	1.34	0.179	1.206 (0.918–1.585)	1.11	0.574	0.00%	1.04	0.296	−4.44	0.141	0.179	0.941	0.363	0.631	0.950	0.995	0.999	0.994	0.999	1.000
**Source of control**																					
HB	15	1.30	0.192	1.229 (0.902–1.674)	85.04	0.000	83.50%	1.39	0.166	2.19	**0.048**	0.191	0.897	0.390	0.657	0.955	0.995	1.000	0.994	0.999	1.000
PB	9	1.69	0.091	1.290 (0.960–1.732)	26.80	0.001	70.10%	0.31	0.754	−0.58	0.577	0.090	0.842	0.243	0.491	0.914	0.991	0.999	0.990	0.999	1.000
**Cancer types**																					
Lung cancer	4	0.98	0.326	1.311 (0.764–2.250)	23.39	0.000	87.20%	0.34	0.734	−0.29	0.802	0.326	0.687	0.587	0.810	0.979	0.998	1.000	0.993	0.999	1.000
Pancreatic caner	3	1.70	0.089	1.577 (0.933–2.664)	5.17	0.075	61.30%	0.00	1.000	0.26	0.841	0.089	0.426	0.384	0.652	0.954	0.995	1.000	0.986	0.999	1.000
Colorectal cancer	3	3.50	0.000	0.693 (0.564–0.851)	0.73	0.686	0.00%	1.04	0.296	1.11	0.466	0.000	0.644	**0.002**	**0.006**	**0.067**	0.419	0.878	**0.592**	0.936	0.999
Gastric cancer	4	0.29	0.774	0.939 (0.610–1.445)	6.43	0.092	54.30%	0.34	0.734	1.59	0.253	0.775	0.940	0.712	0.881	0.988	0.999	1.000	0.996	1.000	1.000
Liver cancer	2	0.80	0.421	2.130 (0.337–13.456)	15.55	0.000	93.60%	0.00	1.000			0.421	0.355	0.781	0.914	0.992	0.999	1.000	0.991	0.999	1.000
Esophageal cancer	2	2.01	0.044	**1.544 (1.011**–**2.359)**	1.49	0.223	32.80%	0.00	1.000			0.045	0.447	0.230	0.473	0.908	0.990	0.999	0.979	0.998	1.000
Colon cancer	2	2.59	0.010	**1.877 (1.166**–**3.054)**	0.03	0.873	0.00%	0.00	1.000			0.011	0.183	**0.155**	0.355	0.858	0.984	0.998	0.943	0.994	1.000
Rectal cancer	2	0.28	0.782	1.057 (0.714–1.563)	0.25	0.620	0.00%	0.00	1.000			0.781	0.960	0.709	0.880	0.988	0.999	1.000	0.997	1.000	1.000
**TT/CT + CC**																					
**Overall**	14	0.09	0.931	1.012 (0.781–1.311)	29.00	0.007	55.20%	0.33	0.743	0.89	0.391	0.928	0.999	0.736	0.893	0.989	0.999	1.000	0.998	1.000	1.000
**Ethnicity**																					
Asian	9	1.42	0.155	0.869 (0.717–1.054)	14.37	0.073	44.30%	0.31	0.754	−0.30	0.772	0.154	0.996	0.317	0.582	0.939	0.994	0.999	0.995	1.000	1.000
Mix	3	2.40	0.017	**1.757 (1.108**–**2.786)**	0.79	0.673	0.00%	0.00	1.000	−0.72	0.602	0.017	0.251	**0.165**	0.373	0.867	0.985	0.998	0.957	0.996	1.000
**Source of control**																					
HB	9	1.79	0.073	0.828 (0.673–1.018)	15.54	0.049	48.50%	0.31	0.754	0.63	0.551	0.073	0.980	**0.183**	0.402	0.881	0.987	0.999	0.991	0.999	1.000
PB	5	1.31	0.190	1.321 (0.871–2.005)	8.36	0.079	52.10%	0.73	0.462	0.91	0.430	0.191	0.725	0.442	0.703	0.963	0.996	1.000	0.992	0.999	1.000
**Cancer types**																					
Lung cancer	4	0.10	0.922	1.033 (0.540–1.977)	11.21	0.011	73.20%	−0.34	1.000	−0.24	0.836	0.922	0.870	0.761	0.905	0.991	0.999	1.000	0.995	0.999	1.000
Pancreatic cancer	2	0.24	0.808	1.062 (0.654–1.725)	0.72	0.395	0.00%	0.00	1.000			0.808	0.919	0.725	0.888	0.989	0.999	1.000	0.996	1.000	1.000
Colorectal cancer	2	2.50	0.012	0.634 (0.444–0.906)	1.17	0.280	14.30%	0.00	1.000			0.012	0.391	**0.086**	0.221	0.758	0.969	0.997	0.950	0.995	1.000
Breast cancer	2	1.71	0.086	1.615 (0.934–2.795)	0.50	0.480	0.00%	0.00	1.000			0.087	0.396	0.397	0.664	0.956	0.995	1.000	0.986	0.999	1.000

Bold value indicates statistically significant OR values, values with FPRP less than 0.2, and values with BFDP less than 0.8.

#### 3.2.2 Relationship between A1298C polymorphism and cancer among smokers, non-smokers, drinkers, and non-drinkers

The analysis of included articles revealed that A1298C polymorphism was irrelevant to overall cancer risk among smokers and non-smokers. However, subgroup analyses revealed a significant increase in cancer risk among the mixed population in the dominant model, with a 1.531-fold increased risk for nonsmokers and a 1.609-fold increased risk for smokers. No significant association between A1298C polymorphism and cancer risk was observed among Asian and Caucasian populations, regardless of smoking status. Detailed results can be found in Table 5.

**Table 5:** Integral analysis of the association between A1298C polymorphism and cancer risk among smoking population

Comparative model	No.	*Z*	*p*	OR (95% CI)	Heterogeneity			*Z*	Begg’s Test	*t*	Egger’s test	FPRP *p*-value^a^	FPRP statistical power^b^	FPRP prior probability					BFDP prior probability		
					Heterogeneity chi-squared	*p*	*I* ^2^							0.25	0.10	0.01	0.001	0.0001	0.01	0.001	0.000010
**smoker**																					
**CC/AA**																					
**Overall**	7	0.23	0.815	0.968 (0.740–1.268)	6.69	0.35	10.40%	1.20	0.230	1.76	0.128	0.813	0.997	0.710	0.880	0.988	0.999	1.000	0.998	1.000	1.000
**Ethnicity**																					
Caucasian	3	0.28	0.777	1.169 (0.398–3.432)	5.34	0.07	62.60%	1.04	0.296	0.77	0.583	0.776	0.675	0.775	0.912	0.991	0.999	1.000	0.993	0.999	1.000
Asian	3	0.79	0.429	1.286 (0.689–2.402)	0.12	0.94	0.00%	0.00	1.000	−0.28	0.824	0.430	0.685	0.653	0.850	0.984	0.998	1.000	0.994	0.999	1.000
**Source of control**																					
HB	2	0.98	0.325	0.857 (0.629–1.166)	0.28	0.60	0.00%	0.00	1.000			0.326	0.945	0.509	0.756	0.972	0.997	1.000	0.996	1.000	1.000
PB	5	1.25	0.211	1.427 (0.817–2.491)	4.97	0.29	19.60%	0.73	0.462	0.83	0.469	0.211	0.570	0.526	0.769	0.973	0.997	1.000	0.992	0.999	1.000
**Cancer type**																					
Lung cancer	3	0.71	0.480	1.556 (0.457–5.306)	5.29	0.07	62.20%	1.04	0.296	1.70	0.339	0.480	0.477	0.751	0.901	0.990	0.999	1.000	0.992	0.999	1.000
**AC/AA**																					
**Overall**	7	0.20	0.838	0.965 (0.684–1.361)	18.76	0.01	68.00%	0.00	1.000	1.85	0.124	0.839	0.982	0.719	0.885	0.988	0.999	1.000	0.997	1.000	1.000
**Ethnicity**																					
Caucasian	3	0.94	0.349	0.781 (0.465–1.311)	4.88	0.09	59.00%	1.04	0.296	1.05	0.485	0.350	0.725	0.591	0.813	0.979	0.998	1.000	0.994	0.999	1.000
Asian	3	1.40	0.162	1.213 (0.926–1.590)	0.02	0.99	0.00%	0.00	1.000	−0.67	0.622	0.162	0.938	0.341	0.608	0.945	0.994	0.999	0.994	0.999	1.000
**Source of control**																					
HB	2	0.45	0.633	0.862 (0.452–1.645)	7.43	0.01	86.50%	0.00	1.000			0.652	0.782	0.714	0.882	0.988	0.999	1.000	0.995	0.999	1.000
PB	5	0.52	0.605	1.076 (0.815–1.422)	4.76	0.31	15.90%	−0.24	1.000	−0.14	0.901	0.607	0.990	0.648	0.846	0.984	0.998	1.000	0.997	1.000	1.000
**Cancer type**																					
Lung cancer	3	0.01	0.993	1.003 (0.502–2.005)	6.63	0.04	69.80%	1.04	0.296	5.23	0.12	0.993	0.873	0.773	0.911	0.991	0.999	1.000	0.995	0.999	1.000
**CC + AC/AA**																					
**Overall**	14	0.52	0.606	1.065 (0.839–1.351)	42.46	0.00	69.40%	0.77	0.443	1.68	0.118	0.604	0.998	0.645	0.845	0.984	0.998	1.000	0.998	1.000	1.000
**Ethnicity**																					
Caucasian	4	0.29	0.770	0.934 (0.592–1.475)	13.96	0.00	78.50%	1.02	0.308	1.34	0.312	0.770	0.926	0.714	0.882	0.988	0.999	1.000	0.996	1.000	1.000
Asian	7	0.51	0.608	1.052 (0.868–1.275)	7.08	0.31	15.20%	1.20	0.230	−0.37	0.727	0.605	1.000	0.645	0.845	0.984	0.998	1.000	0.998	1.000	1.000
Mix	3	2.73	0.006	**1.531 (1.127**–**2.080)**	2.17	0.34	7.90%	1.04	0.296	−17.92	**0.035**	0.006	0.448	**0.041**	**0.115**	0.588	0.935	0.993	0.922	0.992	1.000
**Source of control**																					
HB	6	0.29	0.771	1.057 (0.728–1.535)	28.22	0.00	82.30%	0.00	1.000	2.01	0.115	0.771	0.967	0.705	0.878	0.987	0.999	1.000	0.997	1.000	1.000
PB	8	0.95	0.343	1.102 (0.901–1.348)	9.66	0.21	27.60%	0.37	0.711	−0.51	0.627	0.345	0.999	0.509	0.757	0.972	0.997	1.000	0.997	1.000	1.000
**Cancer type**																					
Lung cancer	3	0.36	0.719	1.158 (0.521–2.573)	9.42	0.01	78.80%	1.04	0.296	3.25	0.19	0.719	0.737	0.745	0.898	0.990	0.999	1.000	0.994	0.999	1.000
Colorectal cancer	3	1.02	0.307	0.853 (0.628–1.158)	1.65	0.44	0.00%	0.00	1.000	0.68	0.618	0.308	0.943	0.495	0.746	0.970	0.997	1.000	0.996	1.000	1.000
Bladder cancer	2	0.41	0.684	0.890 (0.508–1.558)	2.56	0.11	60.90%	0.00	1.000			0.683	0.844	0.708	0.879	0.988	0.999	1.000	0.995	1.000	1.000
**CC/AA + AC**																					
**Overall**	8	0.27	0.79	1.036 (0.800–1.340)	6.34	0.50	0.00%	0.37	0.711	0.21	0.841	0.788	0.998	0.703	0.877	0.987	0.999	1.000	0.998	1.000	1.000
**Ethnicity**																					
Caucasian	3	0.41	0.678	1.065 (0.791–1.433)	3.41	0.18	41.30%	1.04	0.296	0.69	0.614	0.677	0.988	0.673	0.861	0.985	0.999	1.000	0.997	1.000	1.000
Asian	4	0.26	0.794	0.928 (0.531–1.622)	3.1	0.38	3.20%	1.02	0.308	−1.93	0.193	0.793	0.877	0.731	0.891	0.989	0.999	1.000	0.995	1.000	1.000
**Source of control**																					
HB	3	0.37	0.711	0.946 (0.706–1.269)	2.55	0.28	21.60%	1.04	0.296	−1.08	0.474	0.711	0.990	0.683	0.866	0.986	0.999	1.000	0.997	1.000	1.000
PB	5	1.22	0.223	1.405 (0.813–2.430)	3.25	0.52	0.00%	0.73	0.462	1.01	0.388	0.224	0.593	0.531	0.773	0.974	0.997	1.000	0.992	0.999	1.000
**Cancer type**																					
Lung cancer	3	0.57	0.567	1.092 (0.808–1.474)	3.19	0.20	37.30%	1.04	0.296	1.35	0.407	0.565	0.981	0.634	0.838	0.983	0.998	1.000	0.997	1.000	1.000
**non-smoker**																					
**CC/AA**																					
**Overall**	**7**	0.97	0.332	1.239 (0.803–1.912)	3.26	0.78	0.00%	0.30	0.764	−0.14	0.891	0.333	0.806	0.553	0.788	0.976	0.998	1.000	0.994	0.999	1.000
**Ethnicity**																					
Caucasian	3	0.61	0.539	1.223 (0.643–2.328)	0.21	0.90	0.00%	0.00	1.000	0.79	0.575	0.540	0.733	0.688	0.869	0.986	0.999	1.000	0.994	0.999	1.000
Asian	3	1.49	0.136	1.664 (0.852–3.250)	0.31	0.86	0.00%	0.00	1.000	−0.16	0.899	0.136	0.381	0.517	0.763	0.973	0.997	1.000	0.988	0.999	1.000
**Source of control**																					
HB	2	0.41	0.68	1.149 (0.594–2.222)	0.03	0.87	0.00%	0.00	1.000			0.680	0.786	0.722	0.886	0.988	0.999	1.000	0.994	0.999	1.000
PB	5	0.93	0.352	1.314 (0.739–2.334)	3.11	0.54	0.00%	0.24	0.806	−0.31	0.777	0.352	0.674	0.610	0.824	0.981	0.998	1.000	0.993	0.999	1.000
**Cancer type**																					
Lung cancer	3	0.65	0.515	1.244 (0.645–2.397)	0.3	0.86	0.00%	0.00	1.000	1.75	0.33	0.514	0.712	0.684	0.867	0.986	0.999	1.000	0.994	0.999	1.000
**AC/AA**																					
**Overall**	**7**	0.1	0.924	0.975 (0.584–1.628)	24.32	0.00	75.30%	1.20	0.230	−1.83	0.126	0.923	0.927	0.749	0.900	0.990	0.999	1.000	0.996	1.000	1.000
**Ethnicity**																					
Caucasian	3	1.46	0.146	0.569 (0.266–1.216)	5.41	0.07	63.00%	0.00	1.000	−1.73	0.333	0.146	0.341	0.561	0.793	0.977	0.998	1.000	0.988	0.999	1.000
Asian	3	1.84	0.066	1.643(0.967–2.791)	4.47	0.11	55.30%	0.00	1.000	−0.86	0.549	0.066	0.368	0.351	0.618	0.947	0.994	0.999	0.983	0.998	1.000
**Source of control**																					
HB	2	0.48	0.628	1.082 (0.786–1.489)	0.86	0.35	0.00%	0.00	1.000			0.629	0.978	0.659	0.853	0.985	0.998	1.000	0.997	1.000	1.000
PB	5	0.4	0.692	0.829 (0.328–2.095)	21.53	0.00	81.40%	0.73	0.462	−2.12	0.125	0.692	0.678	0.754	0.902	0.990	0.999	1.000	0.993	0.999	1.000
**Cancer type**																					
Lung cancer	3	0.75	0.451	0.858 (0.576–1.278)	1.32	0.52	0.00%	0.00	1.000	−1.42	0.39	0.451	0.893	0.603	0.820	0.980	0.998	1.000	0.996	1.000	1.000
**CC + AC/AA**																					
**Overall**	14	0.2	0.843	1.030 (0.767–1.383)	35.5	0.00	63.40%	1.42	0.155	−2.27	**0.043**	0.844	0.994	0.718	0.884	0.988	0.999	1.000	0.997	1.000	1.000
**Ethnicity**																					
Caucasian	4	1.62	0.104	0.792 (0.597–1.050)	4.09	0.25	26.60%	1.02	0.308	−1.59	0.253	0.105	0.884	0.263	0.517	0.922	0.992	0.999	0.991	0.999	1.000
Asian	7	0.19	0.848	1.047 (0.655–1.673)	18.06	0.01	66.80%	1.20	0.230	−2.81	**0.038**	0.848	0.934	0.731	0.891	0.989	0.999	1.000	0.996	1.000	1.000
Mix	3	2.38	0.018	**1.609 (1.087**–**2.381)**	1.74	0.42	0.00%	0.00	1.000	−0.37	0.777	0.017	0.363	**0.126**	0.301	0.826	0.980	0.998	0.960	0.996	1.000
**Source of control**																					
HB	6	1.57	0.116	1.187 (0.958–1.471)	7.1	0.21	29.50%	0.75	0.452	0.96	0.391	0.117	0.984	0.263	0.518	0.922	0.992	0.999	0.994	0.999	1.000
PB	8	0.9	0.366	0.761 (0.420–1.376)	28.4	0.00	75.30%	0.37	0.711	−2.52	**0.045**	0.366	0.669	0.621	0.831	0.982	0.998	1.000	0.993	0.999	1.000
**Cancer type**																					
Lung cancer	3	0.42	0.676	0.923 (0.632–1.346)	0.33	0.85	0.00%	0.00	1.000	−1.65	0.347	0.677	0.955	0.680	0.865	0.986	0.999	1.000	0.996	1.000	1.000
Colorectal cancer	3	0.18	0.854	0.966 (0.669–1.396)	1.07	0.59	0.00%	0.00	1.000	−0.13	0.92	0.854	0.976	0.724	0.887	0.989	0.999	1.000	0.997	1.000	1.000
Bladder cancer	2	1.43	0.153	0.584 (0.279–1.222)	2.36	0.12	57.70%	0.00	1.000			0.153	0.363	0.559	0.792	0.977	0.998	1.000	0.989	0.999	1.000
**CC/AA + AC**																					
**Overall**	8	0.51	0.612	1.108 (0.745–1.647)	4.34	0.50	0.00%	0.87	0.386	0.11	0.912	0.612	0.933	0.663	0.855	0.985	0.998	1.000	0.996	1.000	1.000
**Ethnicity**																					
Caucasian	3	1.21	0.226	1.458 (0.792–2.683)	1.21	0.55	0.00%	0.00	1.000	1.13	0.461	0.226	0.536	0.558	0.791	0.977	0.998	1.000	0.991	0.999	1.000
Asian	4	0.36	0.719	1.116 (0.613–2.033)	1.8	0.61	0.00%	−0.34	1.000	−0.24	0.836	0.720	0.833	0.722	0.886	0.988	0.999	1.000	0.995	0.999	1.000
**Source of control**																					
HB	3	0.03	0.98	0.992 (0.546–1.804)	1.09	0.58	0.00%	1.04	0.296	−1.61	0.354	0.979	0.904	0.765	0.907	0.991	0.999	1.000	0.995	1.000	1.000
PB	5	0.7	0.482	1.210 (0.711–2.057)	5.08	0.28	21.20%	0.73	0.462	0.59	0.599	0.481	0.786	0.647	0.846	0.984	0.998	1.000	0.995	0.999	1.000
**Cancer type**																					
Lung cancer	3	1.2	0.23	1.460 (0.787–2.709)	1.36	0.51	0.00%	0.00	1.000	1.35	0.406	0.230	0.534	0.564	0.795	0.977	0.998	1.000	0.992	0.999	1.000

Bold value indicates statistically significant OR values, values with FPRP less than 0.2, and values with BFDP less than 0.8.

Similar to the findings for C677T polymorphism, the A1298C variation was associated with an increased cancer risk among non-drinkers in the dominant model. The analysis showed a significant association with a 1.171-fold increased risk in non-drinkers. However, no significant association was observed between A1298C polymorphism and cancer risk among drinkers. Subgroup analyses indicated that the presence of the dominant variant did not correlate with cancer risk among both smokers and non-smokers. Please refer to Table 6 for detailed results.

**Table 6:** Integral analysis of the association between A1298C polymorphism and cancer risk among drinking population

Comparative model	No.	*Z*	*p*	OR (95% CI)	Heterogeneity			*Z*	Begg’s Test	*t*	Egger’s test	FPRP *p*-value^a^	FPRP statistical power^b^	FPRP prior probability					BFDP prior probability		
					Heterogeneity chi-squared	*p*	*I* ^2^							0.25	0.10	0.01	0.001	0.0001	0.01	0.001	0.000010
**Drinker**																					
**CC/AA**																					
**Overall**	5	1.61	0.108	1.305 (0.943–1.807)	1.19	0.88	0.00%	0.24	0.806	−0.09	0.932	0.109	0.799	0.290	0.551	0.931	0.993	0.999	0.991	0.999	1.000
**Ethnicity**																					
Asian	4	0.89	0.374	1.309 (0.723–2.369)	1.19	0.76	0.00%	−0.34	1.000	−0.24	0.836	0.374	0.674	0.625	0.833	0.982	0.998	1.000	0.994	0.999	1.000
**Cancer type**																					
Lung cancer	2	1.29	0.198	1.271 (0.882–1.829)	0.16	0.69	0.00%	0.00	1.000			0.197	0.814	0.420	0.685	0.960	0.996	1.000	0.993	0.999	1.000
**AC/AA**																					
**Overall**	5	0.88	0.377	1.078 (0.913–1.272)	6.11	0.19	34.60%	0.24	0.806	0.28	0.795	0.374	1.000	0.529	0.771	0.974	0.997	1.000	0.998	1.000	1.000
**Ethnicity**																					
Asian	4	0.49	0.626	1.093 (0.765–1.561)	6.11	0.11	50.90%	0.34	0.734	0.46	0.692	0.625	0.959	0.662	0.854	0.985	0.998	1.000	0.997	1.000	1.000
**Cancer type**																					
Lung cancer	2	0.85	0.395	1.093 (0.890–1.342)	0.04	0.85	0.00%	0.00	1.000			0.396	0.999	0.543	0.781	0.975	0.997	1.000	0.997	1.000	1.000
**CC + AC/AA**																					
**Overall**	12	1.65	0.100	1.115 (0.979–1.269)	18.62	0.07	40.90%	0.75	0.451	−0.50	0.625	0.099	1.000	0.229	0.471	0.908	0.990	0.999	0.996	1.000	1.000
**Ethnicity**																					
Asian	9	0.28	0.778	0.974 (0.808–1.173)	10.33	0.24	22.50%	0.52	0.602	0.00	0.999	0.781	1.000	0.701	0.875	0.987	0.999	1.000	0.998	1.000	1.000
**Cancer type**																					
Colon cancer	3	0.99	0.324	1.292 (0.777–2.148)	1.43	0.49	0.00%	0.00	1.000	0.32	0.806	0.323	0.718	0.575	0.802	0.978	0.998	1.000	0.994	0.999	1.000
Colorectal cancer	2	1.88	0.061	0.745 (0.548–1.013)	0.07	0.79	0.00%	0.00	1.000			0.060	0.761	**0.192**	0.417	0.887	0.988	0.999	0.986	0.999	1.000
Lung cancer	2	1.14	0.254	1.121 (0.922–1.263)	0.00	0.98	0.00%	0.00	1.000			0.061	1.000	**0.154**	0.353	0.857	0.984	0.998	0.994	0.999	1.000
Rectal cancer	2	1.25	0.211	0.642 (0.321–1.285)	0.75	0.39	0.00%	0.00	1.000			0.211	0.458	0.580	0.806	0.979	0.998	1.000	0.991	0.999	1.000
**CC/AA + AC**																					
**Overall**	6	1.33	0.183	1.224 (0.909–1.649)	2.34	0.80	0.00%	0.00	1.000	−0.40	0.708	0.184	0.909	0.377	0.645	0.952	0.995	1.000	0.994	0.999	1.000
**Ethnicity**																					
Asian	5	0.61	0.541	1.168 (0.711–1.919)	2.29	0.68	0.00%	0.24	0.806	−0.30	0.784	0.540	0.838	0.659	0.853	0.985	0.998	1.000	0.995	1.000	1.000
**Cancer type**																					
Lung cancer	2	1.13	0.258	1.225 (0.862–1.739)	0.17	0.68	0.00%	0.00	1.000			0.256	0.871	0.469	0.726	0.967	0.997	1.000	0.995	0.999	1.000
**Non-drinker**																					
**CC/AA**																					
**Overall**	5	0.70	0.482	1.152 (0.777–1.706)	1.92	0.75	0.00%	0.73	0.462	−2.55	0.084	0.480	0.906	0.614	0.827	0.981	0.998	1.000	0.996	1.000	1.000
**Ethnicity**																					
Asian	4	0.24	0.810	1.072 (0.610–1.884)	1.88	0.60	0.00%	1.02	0.308	−3.48	0.074	0.809	0.879	0.734	0.892	0.989	0.999	1.000	0.995	1.000	1.000
**Cancer type**																					
Lung cancer	2	0.38	0.707	1.105 (0.657–1.860)	1.10	0.29	9.10%	0.00	1.000			0.707	0.875	0.708	0.879	0.988	0.999	1.000	0.995	1.000	1.000
**AC/AA**																					
**Overall**	5	1.54	0.123	1.169 (0.959–1.425)	5.82	0.21	31.20%	0.24	0.806	−0.90	0.433	0.122	0.993	0.270	0.526	0.924	0.992	0.999	0.994	0.999	1.000
**Ethnicity**																					
Asian	4	0.04	0.970	0.995 (0.772–1.284)	1.93	0.59	0.00%	−0.31	1.000	0.15	0.893	0.969	0.999	0.744	0.897	0.990	0.999	1.000	0.998	1.000	1.000
**Cancer type**																					
Lung cancer	2	0.31	0.759	1.119 (0.547–2.888)	3.37	0.07	70.40%	0.00	1.000			0.816	0.728	0.771	0.910	0.991	0.999	1.000	0.993	0.999	1.000
**CC + AC/AA**																					
**Overall**	12	2.13	0.033	**1.171 (1.012**–**1.354)**	20.68	0.04	46.80%	0.34	0.732	−1.06	0.313	0.033	1.000	**0.090**	0.230	0.766	0.971	0.997	0.988	0.999	1.000
**Ethnicity**																					
Asian	9	0.34	0.735	0.969 (0.806–1.164)	8.55	0.38	6.40%	0.73	0.466	−1.05	0.327	0.736	1.000	0.688	0.869	0.986	0.999	1.000	0.998	1.000	1.000
**Cancer type**																					
Colon cancer	3	0.31	0.755	1.062 (0.726–1.554)	1.16	0.56	0.00%	0.00	1.000	−1.48	0.378	0.757	0.962	0.702	0.876	0.987	0.999	1.000	0.997	1.000	1.000
Colorectal cancer	2	0.24	0.811	1.034 (0.787–1.359)	0.23	0.63	0.00%	0.00	1.000			0.811	0.996	0.709	0.880	0.988	0.999	1.000	0.998	1.000	1.000
Lung cancer	2	0.15	0.883	1.058 (0.501–2.231)	3.90	0.05	74.40%	0.00	1.000			0.882	0.820	0.763	0.906	0.991	0.999	1.000	0.994	0.999	1.000
Rectal cancer	2	0.62	0.537	0.694 (0.271–2.214)	3.62	0.06	72.40%	0.00	1.000			0.537	0.527	0.754	0.902	0.990	0.999	1.000	0.992	0.999	1.000
**CC/AA + AC**																					
**Overall**	6	0.03	0.973	0.994 (0.683–1.446)	3.07	0.69	0.00%	1.50	0.133	−2.16	0.097	0.975	0.982	0.749	0.899	0.990	0.999	1.000	0.997	1.000	1.000
**Ethnicity**																					
Asian	5	0.11	0.911	0.970 (0.570–1.652)	3.10	0.54	0.00%	1.71	0.086	−6.27	**0.008**	0.911	0.916	0.749	0.899	0.990	0.999	1.000	0.996	1.000	1.000
**Cancer type**																					
Lung cancer	2	0.21	0.830	0.946 (0.570–1.569)	0.60	0.44	0.00%	0.00	1.000			0.830	0.912	0.732	0.891	0.989	0.999	1.000	0.996	1.000	1.000

Bold value indicates statistically significant OR values, values with FPRP less than 0.2, and values with BFDP less than 0.8.

### 3.3 Publication bias

Possible publication bias was examined based on the result of the calculation of Begg’s test and Egger’s test. For C677T polymorphism, some results showed publication bias based on Egger’s test values (for smokers: CT + TT vs CC, *p* = 0.009; for non-smokers: TT vs CT + CC, *p* = 0.043; for drinkers: TT vs CC, *p* = 0.002). For A1298C polymorphism (for non-smokers: CC + AC vs AA, *p* = 0.043).

### 3.4 Sensitivity analysis

Only the homozygote comparison of smokers in the sensitivity analysis detected transformation of results for A1298C, indicating the reliability of most of our findings. To explore the sources of heterogeneity, we conducted a meta-regression using appropriate models based on *I* ^2^ and chi-square *p*-values. The meta-regression analysis identified the year of publication and the region of the control group as potential sources of heterogeneity.

### 3.5 FPRP and BFDP tests

The FPRP and BFDP values for the C677T and A1298C polymorphisms are presented in Tables 3–6. The FPRP was utilized to assess the likelihood of significant findings in the results. Based on an OR of 1.5, with a prior probability of 0.25 and 0.1, several values were found to be less than 0.2 in almost all models. However, there were only a few BFDP estimations below 0.8 when considering a prior probability of 0.01, 0.001, and 0.000001 with an OR of 1.5. It is worth noting that only the estimations with FPRP less than 0.2 and BFDP less than 0.8 are deemed significant. These significant estimations suggest that our results should be interpreted cautiously.

## 4 Discussion

The two SNPs of most interest in *MTHFR*, C677T and A1298C, are both thought to be associated with possible carcinogenesis by altering the stability of the *MHTFR* enzyme [61].

Although the correlation between *MTHFR* SNPs and cancer susceptibility has been widely studied, most meta-analyses supported an association between the MHTFR SNPs and an increased risk of cancer [62,63,64]. Here, as possible cancer risk factors, tobacco and alcohol consumption might have direct or indirect synergistic actions on cancers with enzymes regulated by these SNPs. We decided to use this meta-analysis to assess the association of *MTHFR* C677T or A1298C polymorphism alone and in combination with smoking or drinking on cancers.

Chemicals found in tobacco, such as nitrosamines, can cause DNA recombination, a process that can lead to mutations in other cancer related genes, thereby increasing the risk of the disease [65].

Our research suggests that smoking increases the overall risk of cancer in individuals with the C677T variant. However, no such effect was found in carriers of the A1298C variant. This may be related to the loss of enzyme activity caused by MTHFR mutation, where the enzyme activity of A1298C mutant is reduced to a lesser extent than C677T. Individuals carrying the TT genotype of the C677T polymorphism exhibit elevated levels of total homocysteine (tHcy) and reduced levels of folate in their serum compared to those with the CT and CC genotypes [66]. These mutations lead to a reduction in the universal methyl donor SAM during folic acid metabolism, which may result in genomic instability and DNA fragmentation. Under normal conditions, cells maintain a standard methylation pattern by balancing the DNA methylation and demethylation processes. This balance is disrupted under pathological conditions such as inflammation, oxidative stress, and cancer, leading to different phenotypes [67].

Moreover, previous studies have demonstrated that hydrocarbons in tobacco reduce the bioactivity of vitamin B12 and folic acid. Smokers in the National Center for Health Statistics study also had lower levels of red blood cell folate compared to non-smokers [68]. Similarly, a study showed that pregnant women exposed to smoking had lower levels of folic acid compared to women not exposed to tobacco [69].

Furthermore, a significantly elevated risk of carriers harboring the C677T variant was observed in Asian smokers while not in Caucasian smokers, which is consistent with a previous study [62]. This could be attributed to the differences in the frequency of the T allele. Xie et al. in 2015 reported that there were prominent differences in T allele frequencies among Asian (0.396), Indian (0.132), Caucasian (0.326), Middle Eastern (0.201), and African (0.196) populations [63].

Therefore, it is possible that the combined effects of tobacco and C677T on cancers disrupt DNA methylation pathways through folic acid metabolism. However, some researchers have found that the cancer-promoting effect of MTHFR polymorphism may not be influenced by smoking [70,71].

Our study revealed varying combined effects of the C677T polymorphism and smoking on different types of cancer. Interestingly, the role of smoking habits in promoting esophageal cancer risk was similar among both smokers and non-smokers, suggesting that smoking may not be as significant of a factor in esophageal cancer as previously believed. However, it remains an independent factor, as the presence of the C677T polymorphism increased the risk of esophageal cancer in our study, as well as in the meta-analysis conducted by Langevin et al. [72]. Consistent with previous evidence, we also observed that smoking was not a risk factor for colorectal [73] and bladder cancers [74]. The combined effect of tobacco and C677T mutation on liver cancer was the strongest, 1.564 for smokers and 1.072 for nonsmoker. This finding aligns with the meta-analysis conducted by Qi et al. [75], which identified the C677T mutation as a risk factor for liver cancer. In contrast, the situation is different for esophageal cancer, as individuals with the C677T polymorphism who smoke show a significant increase in the risk of liver cancer. This observation can possibly be attributed to the fact that folate metabolism primarily takes place in the liver [76]. An animal study demonstrated that a folate-deficient diet had an impact on gene expression in the liver of offspring mice but not in the colon. Additionally, smoking has been shown to exacerbate folate acid levels. This could be a significant contributing factor to the heightened risk of liver cancer associated with the combination of smoking and the C677T polymorphism. As for A1298C, the majority of studies indicate that it is not associated with an increased risk of cancer and may even have a protective effect against liver cancer [77], gastric cancer [78], and lung cancer [9], which supports the findings of our study, even when considering the influence of smoking. In brief, smoking might be an essential factor in the carcinogenesis in individuals with the C677T mutation, particularly increasing the risk of cancers in Asians, including liver cancer. On the other hand, the combined effect of smoking and A1298C polymorphism on cancers was not significant.

Alcohol is known to act as a folic acid antagonist, and excessive consumption can lead to folate deficiency due to reduced intake of food rich in micronutrients, impaired in intestinal absorption, and changes in metabolic pathways [79]. Thus, we anticipated that alcohol consumption could potentially induce DNA hypomethylation through the aforementioned mechanisms, leading to cancer. When investigating the effect of alcohol consumption on cancer risk, specifically for C677T polymorphism, our findings indicated a decreased risk of cancer among drinkers compared to non-drinkers, particularly for esophageal and colorectal cancer. This finding was contrary to our initial expectations. In a previous study, Taioli et al. showed that the protective effect of C677T variant on colorectal cancer was limited to individuals who regularly consumed alcohol and might be influenced by the folate level [73]. A Japanese case-control study also found a 69% lower risk of esophageal cancer in patients with the TT genotype in the heavy drinking subgroup [53]. Therefore, in addition to alcohol consumption, we suspect that the reduced risk of cancer in drinkers with *MHTFR* variants may be due to ignoring the interaction between folic acid intake and individual *MTHFR* variants. Adequate folate intake could offset the lower enzymatic activity of *MTHFR* that may increase the MTHFR and promote DNA synthesis, while the adequate provision of methyl donors could still be ensured [80]. Plenty of studies have also shown that the protective effect of the TT genotype is limited to individuals with high folic acid intake and low alcohol intake [81,82,83]. However, because of lack of detailed raw data on the association between smoking, drinking, and folic acid intake, we cannot further explore the relationship between *MHTFR* polymorphisms and smoking-folic acid associations or drinking-folic acid associations with cancers. In mixed populations, we observed a lower risk of cancer among drinkers with the C677T variant. For the A1298C polymorphism, we only observed a decreased overall cancer risk in the dominant model. In brief, alcohol consumption may act as a protective factor for cancer incidence in individuals with MTHFR variants. The protective effect appears to be organ-specific and race-specific for the C677T polymorphism, particularly under conditions of adequate folate levels.

When interpreting the results of our meta-analysis, it is important to acknowledge several limitations. First, we focused on studying the individual associations of the C677T and A1298C polymorphisms with cancer risk, without considering their combined effects or the influence of smoking and drinking. However, the available data on the combined effects and interactions between these polymorphisms, as well as smoking and drinking, were insufficient for further analysis. Second, due to limitations in the available data, we were unable to categorize smoking and alcohol consumption into detailed subgroups based on intensity (light/medium/heavy). Instead, we defined individuals as current/ever smokers or non-smokers, as well as drinkers or non-drinkers. This simplified classification may not fully capture the potential nuances of smoking and alcohol consumption patterns.

**Figure 2:**
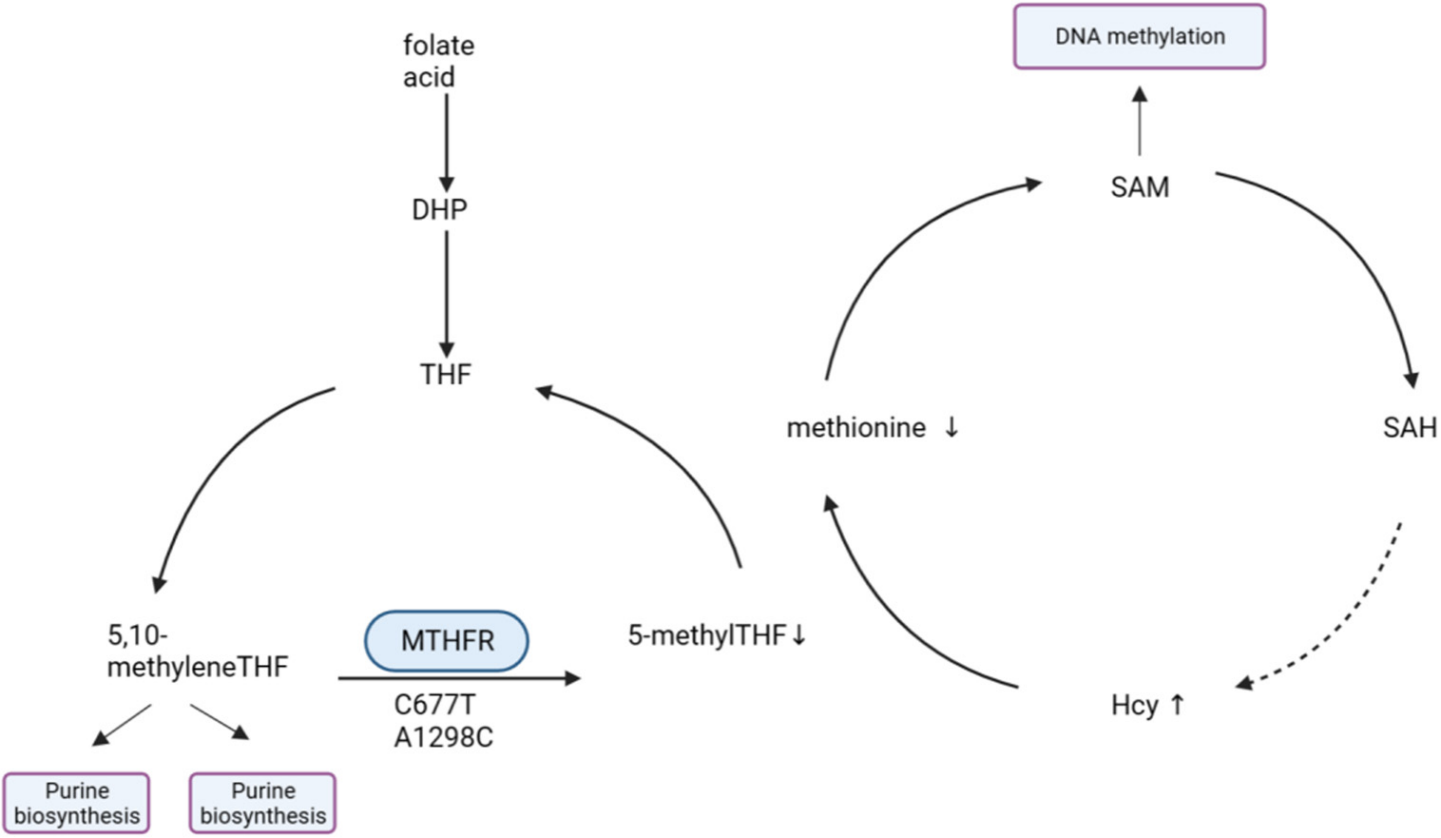
Schematic of folate metabolism. Effects of reduced MTHFR activity on DNA synthesis and methylation. DHF, dihydrofolate; THF, tetrahydrofolate; SAM, S-adenosyl methionine; Hcy, homocysteine; SAH, S-adenosyl homocysteine.

In conclusion, our study highlights the significant influence of smoking and drinking on the relationship between MTHFR polymorphisms and cancer development. The interaction between smoking and the C677T mutation was associated with an increased risk of cancers, particularly liver cancer, and this effect was most prominent in Asian populations. However, the A1298C polymorphism did not show a significant association with cancer risk, even in the presence of tobacco exposure. Conversely, a negative association was observed between alcohol consumption and cancer risk among individuals with either the C677T or A1298C mutation. Future studies with larger sample sizes are needed to further explore the combined effects of tobacco or alcohol and MTHFR polymorphisms at varying folate levels in cancer development (Figure 2).
